# Differential Effects of Pregabalin and Morphine on the Sleep–Wake Cycle and Circadian Rhythms in Mice with Neuropathic Pain

**DOI:** 10.1097/ALN.0000000000005715

**Published:** 2025-08-13

**Authors:** Wenjing Dai, Tommi Kilpeläinen, Manqing Wen, Chandreyee Roy, Anniina Lundén, Maija K. Koskinen, Antti Pertovaara, Anni-Maija Talvio, Henna-Kaisa Wigren, Eija Kalso, Vinko Palada

**Affiliations:** 1 Department of Physiology, SleepWell Research Program, Faculty of Medicine, University of Helsinki, Helsinki, Finland.; 2 Department of Physiology, SleepWell Research Program, Faculty of Medicine, University of Helsinki, Helsinki, Finland.; 3 Department of Physiology, SleepWell Research Program, Faculty of Medicine, University of Helsinki, Helsinki, Finland.; 4 Department of Computer Science, Aalto University School of Science, Espoo, Finland.; 5 Department of Physiology, SleepWell Research Program, Faculty of Medicine, University of Helsinki, Helsinki, Finland.; 6 SleepWell Research Program and Department of Psychology and Logopedics, Faculty of Medicine, University of Helsinki, Helsinki, Finland.; 7 Department of Physiology, Faculty of Medicine, University of Helsinki, Helsinki, Finland.; 8 Department of Pharmacology, Faculty of Medicine, University of Helsinki, Helsinki, Finland.; 9 SleepWell Research Program, Faculty of Medicine, Faculty of Biological and Environmental Sciences, Molecular and Integrative Biosciences Research Program, University of Helsinki, Helsinki, Finland.; 10 Department of Pharmacology, SleepWell Research Program, Faculty of Medicine, University of Helsinki, Helsinki, Finland; Department of Anaesthesiology, Intensive Care and Pain Medicine, Helsinki University Hospital, Helsinki, Finland.; 11 Department of Physiology, SleepWell Research Program, Faculty of Medicine, University of Helsinki, Helsinki, Finland.

## Abstract

**Background:**

Neuropathic pain is commonly associated with disturbances in sleep architecture and circadian rhythms, leading to fragmented sleep, body temperature fluctuations, and altered locomotion. While pregabalin and morphine are frequently prescribed for neuropathic pain management, their effects on sleep and circadian regulation are poorly understood.

**Methods:**

To identify the effects of spared nerve injury (SNI) on sleep architecture and circadian rhythms, male and female C57BL/6JRJ mice were implanted with wireless transmitters for continuous monitoring of electroencephalogram, electromyogram, locomotion, and body temperature. After baseline recordings, SNI was performed, and mechanical and dynamic allodynia was assessed on days 3, 7, and 14 after the surgeries. Pregabalin (11 mg/kg each day) or morphine (6 mg/kg each day) was administered continuously to male mice *via* intraperitoneal osmotic minipumps. Recordings were repeated on postoperative days 7 and 14.

**Results:**

SNI significantly disrupted the sleep–wake cycle by reducing rapid eye movement (REM) sleep duration during the light phase (the habitual sleeping phase for mice) in both sexes and increasing wakefulness in females, without significantly affecting non-REM sleep. Additionally, SNI significantly impaired the circadian rhythmicity of locomotion and body temperature. Pregabalin, but not morphine, significantly restored REM sleep to presurgical levels and restored locomotor activity and body temperature rhythmicity more effectively than morphine. At the molecular level, SNI altered spinal cord circadian gene expression, which pregabalin significantly reversed, whereas morphine showed mixed effects. Furthermore, pregabalin increased sleep spindle occurrence during sleep stage transitions and enhanced the power spectra within the 3.5- to 5.5-Hz range during REM sleep. Morphine did not significantly alter either sleep architecture or microstructure in SNI mice.

**Conclusions:**

Pregabalin, unlike morphine, restores SNI-disrupted sleep architecture, circadian rhythms, and spinal circadian gene expression.

## Abstract 2

In mice, spared nerve injury significantly disrupted sleep–wake cycles, and this was associated with altered spinal cord circadian gene expression. Pregabalin but not morphine reversed both spared nerve injury–induced changes in circadian gene expression and disruption of sleep–wake cycles.

## Editor’s Perspective

**What We Already Know about This Topic**

- Neuropathic pain is commonly associated with disturbances in sleep architecture and circadian rhythms
- Pregabalin and morphine are frequently prescribed for neuropathic pain management, but their effects on sleep and circadian regulation are incompletely understood

**What This Article Tells Us That Is New**

- In mice, spared nerve injury significantly disrupted sleep–wake cycles, and this was associated with altered spinal cord circadian gene expression
- Pregabalin but not morphine reversed both spared nerve injury–induced changes in circadian gene expression and disruption of sleep–wake cycles

Neuropathic pain, caused by nerve injury, is frequently associated with sleep disturbance, significantly impairing health-related quality of life.^1^ Neuropathic pain conditions, such as postherpetic neuralgia,^2^ trigeminal neuralgia,^3^ and diabetic neuropathy,^4^ can severely disrupt sleep quality. In turn, insufficient sleep increases pain intensity, creating a vicious cycle of chronic pain and sleep disorders.^5,6^

Previous studies highlight the profound effects of neuropathic pain on sleep patterns in animal models. Rats with chronic constriction injury exhibit reduced sleep efficiency and increased arousals,^7,8^ while sciatic nerve-ligated mice show more wakefulness and reduced rapid eye movement (REM)^9^ and non-REM (NREM) sleep.^10^ Additionally, neuropathic pain is linked to circadian disruption, affecting the body’s natural 24-h cycle of biological processes, including sleep–wake patterns, hormone release, and body temperature regulation.^11,12^ Patients with diabetic neuropathy and postherpetic neuralgia frequently report peak pain severity during the evening hours.^13^ Furthermore, both pain and sleep are under circadian regulation at the molecular level.^11,14^ These findings suggest a strong inter-relationship between neuropathic pain and circadian rhythms, raising the question of whether analgesics could also alleviate circadian disruptions.

Pregabalin, a structural analog of γ-aminobutyric acid (GABA) that shares structural similarities with gabapentin, is a first-line drug for treating neuropathic pain.^15,16^ Pregabalin exerts its analgesic effects by binding to the α_2_δ_1_ subunit of voltage-gated calcium channels, reducing pronociceptive substance release.^17–19^ Pregabalin reduces sleep interference scores compared with placebo in adults with neuropathic pain^20,21^ and enhances sleep quality in mice with partial sciatic nerve ligation.^22^ However, while evidence supports pregabalin’s role in improving sleep quality, the mechanisms and extent to which it alleviates sleep and circadian disturbances associated with neuropathic pain remain unclear.

Morphine, an opioid analgesic commonly used for cancer pain, is recommended as a third-line drug for neuropathic pain treatment.^16^ Its analgesic mechanism involves binding to μ-opioid receptors, inhibiting the release of excitatory transmitters into synapses in the dorsal horn of the spinal cord and stimulating the production of inhibitory neurotransmitters in the descending pain pathway.^23–25^ Notably, opioids have been shown to adversely affect sleep and circadian rhythms. Morphine reduces deep sleep and REM sleep in healthy human adults^26,27^ and promotes wakefulness in healthy rodents,^28–30^ while fentanyl induces phase shifts of circadian rhythms^31^ and disrupts diurnal activity in rodents.^32^ However, it remains unclear whether morphine exacerbates or alleviates sleep and circadian abnormalities in neuropathic pain.

This study aimed to identify the effects of spared nerve injury (SNI) on sleep architecture and circadian rhythms in mice^33^ and to examine how these effects are influenced by continuous administration of pregabalin and morphine. Based on previous studies, we hypothesized that pregabalin would enhance sleep and protect against circadian disruptions, whereas morphine would not. To minimize sleep–wake disturbances from repeated drug administration, we used osmotic pumps for continuous drug delivery,^34,35^ ensuring consistent dosage while minimizing injection-induced stress. Additionally, a wireless telemetry system enabled free untethered movement of mice and simultaneous recording of electroencephalogram (EEG), electromyogram (EMG), body temperature, and locomotion. This integrated approach allowed us to provide detailed insights into the interplay between neuropathic pain, sleep and circadian rhythms.

## Materials and Methods

### Animals

Wild-type C57BL/6JRJ mice (12 ± 2 weeks old, 25 ± 2 g) were housed in standard animal facilities under controlled conditions (ambient temperature at 22° ± 2°C, humidity at 50 ± 15%, lights on 6:00 am, and lights off 6:00 pm). The mice were provided free access to Teklad 2916 rodent chow (Envigo, United Kingdom) and water. Non-EEG mice were group housed, while EEG-implanted mice were single housed to prevent interference with the recording equipment. To minimize potential environmental confounders, cage positions were randomly assigned and rotated weekly within the room to avoid systematic location effects. All animal procedures complied with the Finnish Act on the Protection of Animals Used for Science or Educational Purposes (497/2013) and Directive 2010/63/EU of the European Parliament and of the Council. Ethical approvals were obtained from the Regional State Administrative Agency for Southern Finland (ESAVI/36258/2020 and ESAVI/30956/2022).

### Experimental Protocols

**Fig. 1.**
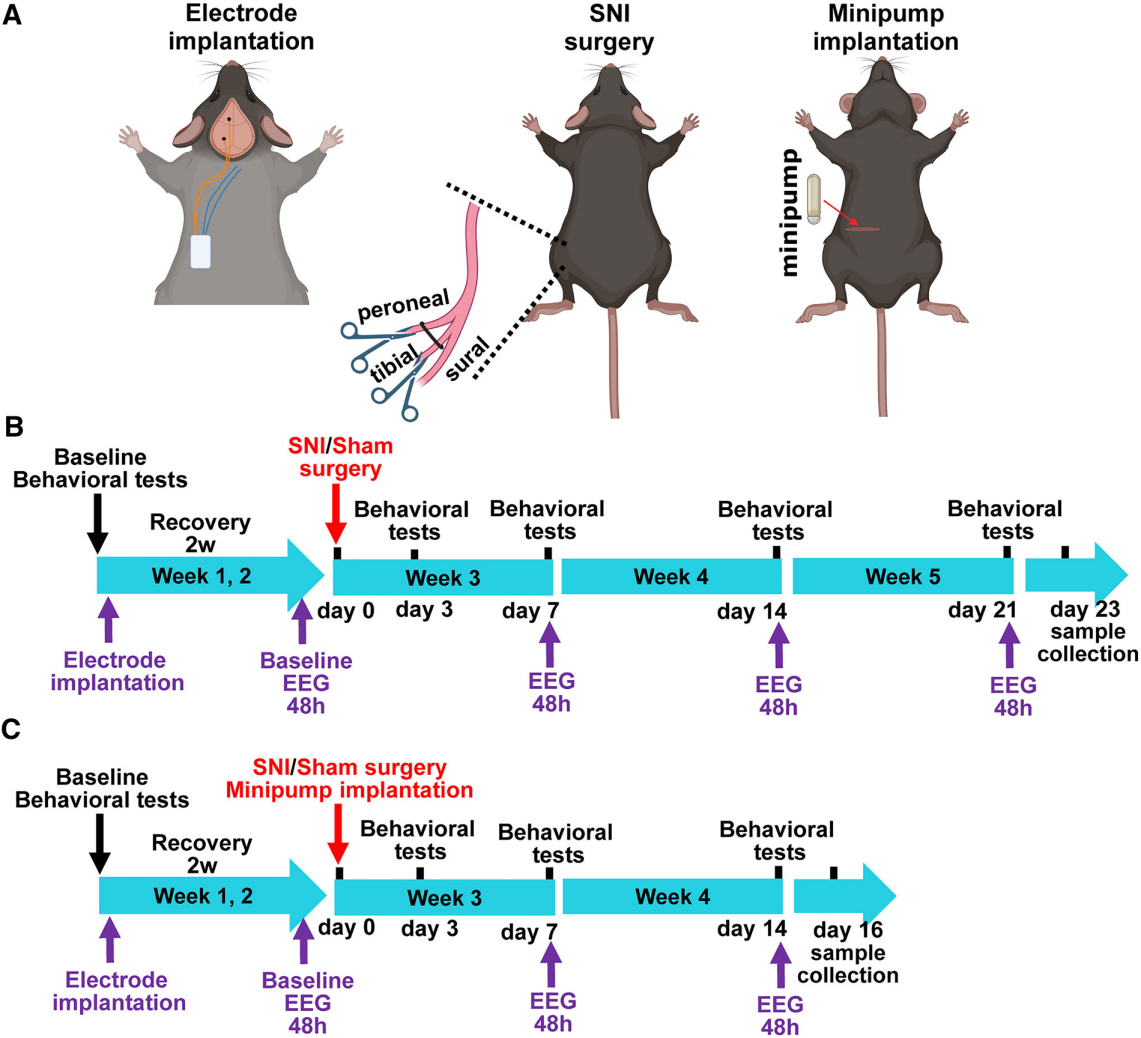
Experimental protocols. (*A*) Schematic diagrams illustrating the implantation of transmitter electrodes, SNI surgery, and minipump implantation in mice (from *left* to *right*). (*B*) Overview of experimental protocol 1. All 46 mice underwent baseline behavioral testing, followed by implantation of EEG transmitter electrodes in the EEG group and 2 weeks of recovery. During the recovery, behavioral tests were conducted on days 3, 8, and 13 after implantation to confirm that the pain-related sensory thresholds had returned to baseline levels before the baseline EEG recording (corresponding data shown in supplemental fig. 1, https://links.lww.com/ALN/E179). Baseline EEG recordings were then performed continuously for 48 h before SNI or sham surgery, which was designated as day 0. Behavioral tests (von Frey and brush tests) for both EEG and non-EEG groups were performed on days 3, 7, 14, and 21 upon SNI or sham surgery. EEG recordings were performed on days 7, 14, and 21 after surgery, with behavioral tests conducted 24 h before the corresponding EEG recordings. (*C*) Overview of experimental protocol 2. A total of 63 mice underwent baseline behavioral testing, followed by transmitter electrode implantation in the EEG group and a 2-week recovery period. Behavioral tests were conducted during the recovery period on days 3, 8, and 13 after implantation to ensure the recovery of pain-related sensory thresholds before baseline EEG recording (corresponding data shown in supplemental fig. 3, https://links.lww.com/ALN/E181). Baseline EEG recordings were conducted continuously for 48 h before SNI or sham surgery (day 0), following the same procedure as in protocol 1. Moreover, in this protocol, osmotic minipumps for drug delivery were implanted immediately after SNI or sham surgery. Behavioral tests (von Frey and brush tests) were conducted on days 3, 7, and 14 after SNI or sham surgery. EEG recordings were conducted on days 7 and 14 after surgery, with behavioral tests performed 24 h before each recording. EEG, electroencephalogram; SNI, spared nerve injury; w, week.

The study was performed using two experimental protocols. To minimize selection bias, the animals were randomly assigned to experimental groups using a computer-generated randomization schedule. Randomization was conducted separately for each protocol by an independent researcher who was not involved in behavioral testing, EEG recording, or data analysis. In the first protocol, 46 mice (23 males and 23 females) were divided into four groups: non-EEG sham (6 males, 6 females); non-EEG SNI (6 males, 6 females); EEG sham (4 males, 4 females); and EEG SNI (7 males, 7 females). All mice underwent two baseline behavioral testing sessions after habituation, and the average value was used as baseline. EEG mice then underwent implantation of transmitter electrodes, followed by a 2-week recovery period. During this period, additional behavioral tests were conducted on days 3, 8, and 13 after implantation (labeled as day −11, day −6, and day −1, respectively, to align with protocol timings). Baseline EEG recordings were performed at the end of the recovery period when the sensory thresholds had returned to preimplantation baseline levels. SNI or sham surgeries were performed 14 days after EEG implantation (designated as day 0). Postsurgical behavioral tests were performed on days 3, 7, 14, and 21, and EEG recordings were conducted at baseline before SNI and on days 7, 14, and 21 after SNI, with behavioral tests performed 24 h before the corresponding EEG recordings (fig. 1B). For non-EEG mice, behavioral testing followed the same surgery-based timeline. The second protocol was performed with 63 male mice divided into six groups: non-EEG sham vehicle (n = 5); non-EEG SNI vehicle (n = 18); non-EEG SNI pregabalin (n = 10); EEG SNI vehicle (n = 15); EEG SNI pregabalin (n = 7); and EEG SNI morphine (n = 8). Based on the results of the first protocol, which revealed similar changes in sleep and circadian rhythms between the sexes upon injury and to minimize variability and to adhere to the 3R principles, we used only male mice in the second protocol. Separate cohorts were used for each protocol to ensure independent groups. In the second protocol, osmotic minipumps were implanted immediately after SNI or sham surgery. Behavioral tests were conducted at baseline before nerve injury and on days 3, 7, and 14 after surgery, and EEG recordings were performed at baseline and on days 7 and 14 after surgery (fig. 1C). Due to the constraint of the osmotic pumps in maintaining consistent drug delivery for up to 16 days, the second protocol was conducted until day 14, instead of day 21 as in the first protocol. In both protocols, all surgical procedures (*e.g.*, SNI, sham, and transmitter or pump implantations) were performed in a randomized order to reduce potential batch effects. The outcome assessments, including behavioral testing, EEG data scoring and analysis, and quantitated polymerase chain reaction (PCR) assays, were performed by investigators blinded to group allocation. Final statistical analyses were conducted using anonymized data sets to minimize potential bias.

### Spared Nerve Injury Model of Neuropathic Pain

SNI surgery was used to induce neuropathic pain in mice by creating a unilateral sciatic nerve injury in the left limb. Before surgery, mice received subcutaneous injections of carprofen (5 mg/kg, Rimadyl; Zoetis Animal Health, Finland) as a preoperative analgesic. Anesthesia was induced by placing the mouse in a chamber with 3.0 to 5.0% isoflurane (Attane Vet; Piramal Critical Care B.V., The Netherlands) mixed with oxygen and maintained using a face mask to deliver 1.5 to 2.5% isoflurane mixed with oxygen throughout the surgery. The surgical procedures were adapted from a study by Richner *et al.*^36^ and involved a 1-cm longitudinal incision proximal to the knee. The tibial and common peroneal nerves were tightly ligated with a 6-0 suture and subsequently axotomized, while preserving the integrity of the sural nerve. The distal nerve stump was sectioned, removing 1 ± 0.2 mm of nerve tissue. The sham procedure involved the same surgical incision without ligating or transecting the nerves. The muscle layer was then carefully closed, and the skin was sutured with surgical knots.

### Implantation of Osmotic Minipumps and Drug Administration

Pregabalin (Orion, Finland) and morphine hydrochloride (University Pharmacy, Finland) were dissolved in sterile 0.9% saline (pH 7.0 to 7.3). The drugs were administered using osmotic minipumps (Alzet model 1002; DURECT Corporation, USA; mean pumping rate, 0.25 µl/h; reservoir volume, 101.5 µl)^34,37^ at dosages of 11 mg/kg each day for pregabalin and 6 mg/kg each day for morphine. Doses were selected based on previous studies demonstrating efficacy in mouse neuropathic pain models^38,39^ and a pilot study (n = 4 SNI mice per group) confirming significant analgesia. Concentrations (pregabalin, 55 mg/ml; morphine, 30 mg/ml) were calculated for a 25- to 30-g mouse to deliver the target dose greater than 14 days, based on the pumping rate of the minipump model.

Empty osmotic minipumps, equipped with flow moderators, were weighed before filling. Pregabalin or morphine solutions were carefully perfused into the pumps, and the exact amount of solution was determined by measuring the weight of the pumps to ensure that the filled volume was greater than 90% of the total capacity (101.5 µl). To avoid batch-related bias, the order of pump implantation across treatment groups was randomized. Subsequently, the mice were anesthetized as mentioned above in the supine position using a mask with 4% isoflurane. After shaving the abdominal hair, the area was sterilized with povidone iodine. An approximately 0.8-cm-long incision was made in the abdominal skin using a scalpel, carefully lifting the musculoperitoneal layer to avoid damage to the bowel. The peritoneal wall was incised directly beneath the cutaneous incision. The filled pump was inserted, the delivery portal first, into the peritoneal cavity using tweezers. The muscle layer and skin were closed with 6-0 sutures, and the area was sterilized. All procedures were performed in a sterile environment immediately after the SNI surgeries. After regaining consciousness, the mice were returned to their housing.

### Behavioral Tests

Before the behavioral testing, the mice were habituated to the experimental room and testing devices for 60 min per day for 2 consecutive days to familiarize them with the testing environment. All behavioral tests were consistently performed during the same daytime window (between 9:00 am and 12:00 pm). Each behavioral session involved a randomized testing order across animals and apparatus, and the experimenters conducting the tests were blinded to group allocation.

#### Paw Withdrawal Threshold/von Frey Test

The von Frey filament test was used to evaluate mechanical allodynia.^36,40^ Each mouse was individually placed in a red translucent plastic cylinder (radius, 5 cm; height, 12 cm) for approximately 30 min for habituation before testing. Filaments, with a range of forces of 0.02, 0.04, 0.07, 0.16, 0.4, 0.6, 1.0, 1.4, and 2.0 g, were manually applied to the lateral part of the paw with increasing force stimuli and bent at a 90° angle. The test was repeated five times with intervals of at least 10 s. A positive reaction was recorded when the mouse responded to at least three out of five stimuli. The filaments above the threshold were applied to validate the threshold. A positive response was defined by brisk paw withdrawal, flinching, licking, or shaking.

#### Dynamic Brush Test

Dynamic mechanical hypersensitivity was evaluated by lightly stroking the external lateral side of the hind paw from heel to toe using a 5/0 paintbrush at a velocity of approximately 2 cm/s.^41^ In naive mice, a typical response is scored as 0, characterized by a rapid movement during which the stimulated paw is lifted for less than 1 s. However, after nerve injury, different pain-related responses were observed. These included sustained lifting of the stimulated paw toward the body for more than 2 s or a single gentle flinching of the stimulated paw, which were scored as 1. A strong lateral paw lift above the level of the body, resembling an exaggerated hind paw withdrawal or a startle-like jump, was scored as 2. Multiple flinching responses or licking of the affected paw were scored as 3. The stimulation was repeated three times at intervals of at least 3 min, and an average score was calculated for each mouse.

### Polysomnography with Telemetric EEG/EMG Recording

For the EEG group, each mouse was single-housed and prepared as follows. Before surgery, the mice received preoperative analgesic carprofen (10 mg/kg Rimadyl; Zoetis Animal Health). Lidocaine (Orion) was locally applied at the incision site for pain management. Anesthesia was induced using 4% isoflurane in a chamber, followed by maintenance with 1.5 to 2% isoflurane while the mouse was positioned in a stereotaxic apparatus for transmitter implantation (HDX02, Data Sciences International, USA).^42^ To minimize operator or order-related confounders, the electrode implantation procedures were performed in randomized order across experimental groups. Postoperatively, carprofen (5 mg/kg) was administered daily for 2 days to aid recovery.

For EEG recording, two biopotential leads were placed in the left epidural space of the frontal region (0.5 mm lateral from midlinel; 1.0 mm anterior to the bregma) and the parietal area (1.5 mm lateral to midline; 3.4 mm posterior from the bregma).^43^ The leads were fixed to the skull with dental cement (GC FujiCEM 2; Plandent, Finland), and two EMG leads were inserted into the cervical trapezius muscle, approximately 1 to 2 mm apart along the same bundle of muscle fibers with a 20-gauge syringe tip (BD Microlance; Thermo Fisher Scientific, USA). Once the dental cement had hardened, the incision was closed with silk sutures (6-0 coated Vicryl Plus; Ethicon, USA). After surgery, the mice underwent a 2-week postoperative recovery period to ensure complete recovery. At the end of the recovery period, baseline EEG/EMG recording was initiated for 48 h at a sampling rate of 500 Hz and a bandwidth range of 0.5 to 80 Hz. The Data Sciences International system, consisting of PhysioTel Receiver plates (model RPC-1) beneath the cages, was used to capture telemetric signals from the implanted transmitters. The communication between implants, receiver plates, and acquisition software was managed by a DSI Matrix 2.0. The DSI Talker interface and Spike2 software (version 10.24; Cambridge Electronic Design, United Kingdom) were used for data recording.

### Sleep Scoring and Analysis of Sleep Spindles, Sleep–Wake Stage Transitions, and EEG Power Spectrum

EEG recordings were divided into 4-s epochs using a manual sleep scoring script in Spike2 (sleepscore_v1.86.s2s; Cambridge Electronic Design). Each epoch was classified into distinct stages—wakefulness, NREM sleep, REM sleep, or noise—based on previously established criteria.^44^ Briefly, wakefulness was identified by low-amplitude, high-frequency EEG activity (greater than 10 Hz) and high EMG activity. NREM sleep was characterized by high-amplitude delta waves (0.5 to 4 Hz) in the EEG signal and low EMG activity, whereas REM sleep was identified by low-amplitude theta waves (5 to 9 Hz) in the EEG, with minimal or absent EMG activity. The total number of epochs for each stage (wakefulness, NREM sleep, and REM sleep) was calculated over the 48-h recording period, and the duration of each stage (in hours) was obtained by multiplying the number of epochs by 4 and dividing the results by 3,600 (number of seconds in 1 h). To account for interindividual variability, the counts were normalized to the baseline level of each mouse, defined as the sleep–wake distribution for each stage recorded for a 48-h period before SNI was performed.

Spindle events were automatically detected using a MATLAB-based script (Spindle_dtect_v17_5s).^45^ For spindle detection, the EEG signal was bandpass-filtered in the sigma range (10 to 15 Hz) using a Butterworth filter. Subsequently, the root-mean-squared (rms) power of the filtered EEG data was computed, using a 750-ms window to smooth the trace and generate a signal envelope; rms values were then cubed to enhance the separation between noise and signal, facilitating the placement of thresholds. Finally, a two-threshold approach was used to establish inclusion criteria for spindle detection, the threshold values being derived from the mean cubed rms transform value of the entire trace (all behavioral states) and including both a lower threshold (1.2 × mean cubed rms, default) and upper threshold (3.5 × mean cubed rms, default). This algorithm was employed to quantify both the total number of spindles and the number occurring during the transitions between wakefulness, NREM sleep, and REM sleep.

To determine the relative contribution of each type of state transition, the number of transitions between each pair of sleep–wake stages (NREM to REM, REM to NREM, wake to NREM, and NREM to wake) was calculated for each animal. These were then expressed as proportions of the total number of state transitions observed in that animal during the recording period.^46^

For EEG power spectral analysis, stage-specific EEG signals were analyzed, using Spike2 software (version 10.24; Cambridge Electronic Design). Power spectra for each sleep state were calculated across a frequency range of 0.5 to 34.5 Hz and 30 to 50 Hz using fast Fourier transform with a 1,024-point size and a Hanning window, providing a 0.5-Hz resolution.^47^ This analysis covered a 24-h recording period, with data collected at 0.5-Hz frequency intervals. Analyses were conducted separately for the light and dark phases, comparing the SNI vehicle group with the pregabalin- or morphine-treated nerve injury groups for each 0.5-Hz interval.

### Locomotor Activity and Body Temperature Analysis

A cosinor analysis was performed to examine the circadian patterns of body temperature and locomotion activity signals, comparing the changes in three circadian parameters: midline estimating statistic of rhythm (mesor), amplitude (the difference between the peak and the mean value of the wave; *i.e.*, mesor), and acrophase (the phase during a cycle when the peak of a rhythm occurs). These parameters were compared between vehicle-treated, pregabalin-treated, and morphine-treated SNI mice on days 7 and 14, relative to baseline (day 0), over a 24-h period. The analysis was conducted using the CircaCompare^48^ and cosinor^49^ packages in R studio. The data were fitted to the standard cosinusoidal model:


Y=k+αcos[τ(t−φ)]+ε (1)

where Y represents the response variable (locomotion or body temperature), k is the mesor, α is the amplitude, τ is the period, φ represents the phase shift from time t = 0 in radian hours, and ε is the random error accounting for variations in Y. The period was fixed at 24 h.

To compare two groups, the following model was used:


Y=k+k1X+(α+α1X)cos[t−(φ+φ1X)]+ε (2)

where X is a dummy variable that represents the different groups. The CircaCompare package reports the estimated parameters k, k_1_, α, α_1_, φ, and φ_1_, along with their standard errors and 95% CI. k_1_, α_1_, and φ_1_ represent the difference in mesor, amplitude, and phase, respectively, between the two groups.

To explore the influence of vigilance states on circadian body temperature rhythms, we further performed stratified cosinor analyses by segmenting the body temperature data for different vigilance states (wakefulness, NREM sleep, and REM sleep). Body temperature values from each state were extracted separately and analyzed over the 24-h light–dark cycle using the same cosinor model described above. This approach enabled state-specific examination of circadian rhythmicity in temperature.

### RNA Extraction and Real-Time Reverse Transcription Quantitative PCR

Upon completion of the experimental protocols, the mice were euthanized with an overdose of isoflurane followed by cervical dislocation, and ipsilateral spinal cord (iSC) tissue was collected on ice. To investigate the diurnal variation in gene expression, mice from each group were randomly assigned to either a morning (9:00 to 11:00 am) or an afternoon group (2:00 to 4:00 pm) for dissection. Total RNA was extracted using TRIzol reagent (Thermo Fisher Scientific). The quantity and purity of the extracted RNA were assessed using a NanoDrop 2000/2000c Spectrophotometer (Thermo Fisher Scientific). The 260/280 ratios, indicating minimal protein contamination, varied from 1.8 to 2.0, while the 260/230 ratios, indicating minimal contamination by chemicals or organic compounds, ranged from 1.8 to 2.2.

A high-capacity complementary DNA reverse transcription kit (Thermo Fisher Scientific) was used, following the manufacturer’s user guide. RNA samples, along with 2× reverse transcription master mix, were placed in a thermal cycler (Bio-Rad, USA) to synthesize first-strand complementary DNA from 1 µg total RNA.

Quantitative real-time PCR was performed with the QuantStudio 5 real-time PCR instrument (Thermo Fisher Scientific), using TaqMan Fast PCR Master Mix (Thermo Fisher Scientific). The spinal expression of the α_2_δ_1_ subunit of the voltage-gated Ca^2+^ channel, the primary target of pregabalin, was assessed, using the TaqMan assay of *Cacna2d1* (Mm00486607_m1), in mice treated with pregabalin and in controls. The spinal expression levels of opioid precursors were measured using *Penk* (Mm01212875_m1) and *Pdyn* (Mm00457573_m1) TaqMan assays, while opioid receptor expression was analyzed with *Oprd1* (Mm01180757_m1), *Oprk1* (Mm01230885_m1), and *Oprm1* (Mm01188089_m1) assays in the mice treated with morphine and in controls. To investigate the impact of SNI and drug administration on the circadian transcriptome, the expression of core circadian genes (*Clock* [Mm00455950_m1], *Bmal1* [Mm00500223_m1], *Cry1* [Mm00514392_m1], *Cry2* [Mm01331539_m1], *Per1* [Mm00501813_m1], *Per2* [Mm01285623_m1], and *Per3* [Mm00478120_m1]) was measured in iSC from sham control mice, SNI mice, pregabalin-treated SNI mice, and morphine-treated mice. The cycle threshold (Ct) values were calculated using the Design & Analysis Software version 2.6 (Thermo Fisher Scientific). Relative gene expression was analyzed using the 2^−ΔCt^ method, with normalization against the *Hprt* (Mm01318746_g1) as the reference gene.

### Statistical Analysis

All experiments were performed with side-by-side controls, and behavioral tests were conducted and analyzed in a double-blind manner. Since no animal deaths occurred during any described procedure, all collected data were included in the analysis. Sample sizes were determined based on the previous publications on behavior testing, EEG, and circadian rhythms analysis.^33,38,39,45,50,51^ The results of behavioral testing were analyzed with two-way analysis of variance (ANOVA) with group and time as factors, followed by Tukey’s multiple *post hoc* comparisons. The data of sleep architecture, spindles, power spectra, and quantitative PCR were tested for normality and lognormality using the Shapiro–Wilk normality test. For variables that had large *P* values for the Shapiro–Wilk test, they were analyzed using Student’s unpaired *t* test. Otherwise, the data sets were analyzed using nonparametric Mann–Whitney test. The results and plots were created with GraphPad Prism (version 9.2.0; GraphPad, USA) and presented as means ± SEM, with the significance level at *P* < 0.05.

## Results

### Painful Nerve Injury Induces Mechanical Allodynia in Male and Female Mice

During the 2-week recovery period after implantation of EEG transmitters, behavioral tests were conducted, confirming that sensory thresholds had returned back to baseline before the surgeries (supplemental fig. 1, https://links.lww.com/ALN/E179). SNI and sham surgeries were performed 14 days after the implantation of telemetric transmitters in the EEG groups, while nerve injury surgeries were conducted concurrently in the non-EEG groups. Mechanical allodynia was assessed using von Frey filament and brush tests on the lateral plantar surface of the ipsilateral hind paw in both male and female mice on days 3, 7, 14, and 21 after surgery.

**Fig. 2.**
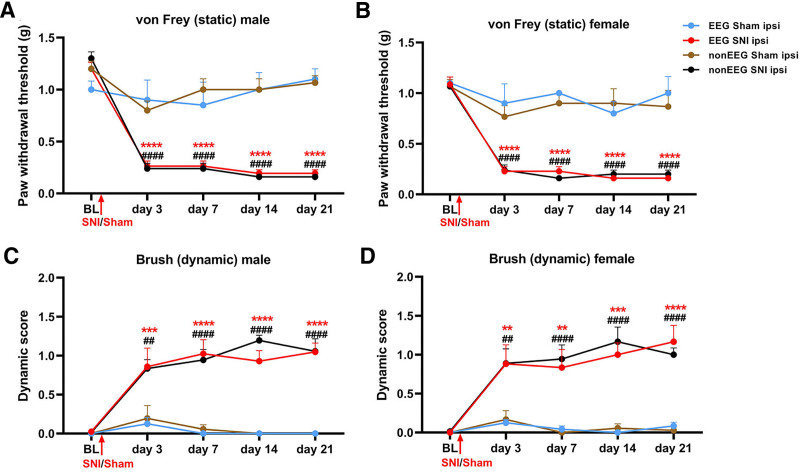
Spared nerve injury (SNI) induces mechanical allodynia under EEG and non-EEG conditions. (*A*) Decreased withdrawal threshold to von Frey filament stimulations in male mice. Significant reductions were observed from day 3 after SNI when comparing SNI mice with sham mice and non-EEG SNI mice with non-EEG sham controls. For EEG sham *versus* EEG SNI: day 3, 0.90 ± 0.191 g *versus* 0.26 ± 0.048 g, *P* < 0.0001, 95% CI, −0.9496 to −0.3246; day 7, 0.85 ± 0.22 g *versus* 0.26 ± 0.048 g, *P* < 0.0001, 95% CI, −0.8996 to −0.2746; day 14, 1.00 ± 0.163 g *versus* 0.19 ± 0.03 g, *P* < 0.0001, 95% CI, −1.118 to −0.4932; and day 21, 1.1 ± 0.10 g *versus* 0.19 ± 0.03 g, *P* < 0.0001, 95% CI, −1.218 to −0.5932. For non-EEG sham *versus* non-EEG SNI: day 3, 0.8 ± 0.08 g *versus* 0.24 ± 0.05 g, *P* < 0.0001, 95% CI, −0.8479 to −0.2721; day 7, 1.00 ± 0.10 g *versus* 0.24 ± 0.05 g, *P* < 0.0001, 95% CI, −1.048 to −0.4721; day 14, 1.00 ± 0.10 g *versus* 0.16 ± 0.00 g, *P* < 0.0001, 95% CI, −1.128 to −0.5521; and day 21, 1.07 ± 0.06 g *versus* 0.16 ± 0.00 g, *P* < 0.0001, 95% CI, −1.195 to −0.6188. For two-way ANOVA, group, F (3, 95) = 85.73, *P* < 0.0001; time, F (4, 95) = 42.05, *P* < 0.0001; and interaction, F (12, 95) = 9.159, *P* < 0.0001. (*B*) Decreased withdrawal threshold to von Frey filament stimulations in female mice. Significant reductions were observed on days 3, 14, and 21 after SNI in the EEG SNI mice compared with the EEG sham controls. Similarly, significant reductions were observed from day 3 after SNI in the non-EEG SNI mice compared with the non-EEG sham. For two-way ANOVA, group, F (3, 95) = 72.66, *P* < 0.0001; time, F (4, 95) = 32.18, *P* < 0.0001; and interaction, F (12, 95) = 4.915, *P* < 0.0001. For EEG sham *versus* EEG SNI: day 3, 0.90 ± 0.19 g *versus* 0.23 ± 0.04 g, *P* < 0.0001, 95% CI, −0.9975 to −0.3454; day 7, 1.00 ± 0.00 g *versus* 0.23 ± 0.04 g, *P* < 0.0001, 95% CI, −1.097 to −0.4454; day 14, 0.80 ± 0.11 g *versus* 0.16 ± 0.00 g, *P* < 0.0001, 95% CI, −0.9661 to −0.3139; and day 21, 1.00 ± 0.16 g *versus* 0.16 ± 0.00 g, *P* < 0.0001, 95% CI, −1.166 to −0.5139. For non-EEG sham *versus* non-EEG SNI: day 3, 0.77 ± 0.10 g *versus* 0.24 ± 0.05 g, *P* < 0.0001, 95% CI, −0.8270 to −0.2263; day 7, 0.90 ± 0.10 g *versus* 0.16 ± 0.00 g, *P* < 0.0001, 95% CI, −1.040 to −0.4397; day 14, 0.90 ± 0.14 g *versus* 0.20 ± 0.20 g, *P* < 0.0001, 95% CI, −1.000 to −0.3997; and day 21, 0.87 ± 0.16 g *versus* 0.20 ± 0.20 g, *P* < 0.0001, 95% CI, −0.9670 to −0.3663. (*C*) Increased dynamic scores in response to brush stimulations in male mice were evident from day 3 after SNI in both the SNI groups, compared with their respective sham groups. For EEG sham *versus* EEG SNI: day 3, 0.13 ± 0.08 *versus* 0.86 ± 0.24, *P* < 0.001, 95% CI, 0.2595 to 1.205; day 7, 0 ± 0.00 *versus* 1.02 ± 0.18, *P* < 0.0001, 95% CI, 0.5512 to 1.496; day 14, 0 ± 0.00 *versus* 0.93 ± 0.13, *P* < 0.0001, 95% CI, 0.4560 to 1.401; and day 21, 0 ± 0.00 g *versus* 1.05 ± 0.11, *P* < 0.0001, 95% CI, 0.5750 to 1.520. For non-EEG sham *versus* non-EEG SNI: day 3, 0.19 ± 0.16 *versus* 0.83 ± 0.11, *P* < 0.01, 95% CI, 0.2035 to 1.074; day 7, 0.06 ± 0.06 *versus* 0.94 ± 0.13, *P* < 0.0001, 95% CI, 0.4535 to 1.324; day 14, 0 ± 0.00 *versus* 1.19 ± 0.06, *P* < 0.0001, 95% CI, 0.7591 to 1.630; and day 21, 0 ± 0.00 *versus* 1.05 ± 0.15, *P* < 0.0001, 95% CI, 0.6202 to 1.491. For two-way ANOVA, group, F (3, 95) = 64.01, *P* < 0.0001; and time, F (4, 95) = 13.65, *P* < 0.0001; interaction, F (12, 95) = 4.56, *P* < 0.0001. (*D*) Increased dynamic scores in response to brush stimulations in female mice were observed from day 3 after SNI in the SNI groups compared with their respective sham controls. For EEG sham *versus* EEG SNI: day 3, 0.12 ± 0.04 *versus* 0.89 ± 0.24, *P* < 0.01, 95% CI, 0.1864 to 1.326; day 7, 0.04 ± 0.04 *versus* 0.83 ± 0.23, *P* < 0.01, 95% CI, 0.2221 to 1.361; day 14, 0 ± 0.00 *versus* 1.00 ± 0.15, *P* < 0.0001, 95% CI, 0.4304 to 1.570; and day 21, 0.08 ± 0.04 *versus* 1.17 ± 0.20, *P* < 0.0001, 95% CI, 0.5138 to 1.653. For non-EEG sham *versus* non-EEG SNI: day 3, 0.17 ± 0.11 *versus* 0.89 ± 0.18, *P* < 0.01, 95% CI, 0.1976 to 1.247; day 7, 0 ± 0.00 *versus* 0.94 ± 0.18, *P* < 0.0001, 95% CI, 0.4198 to 1.469; day 14, 0.05 ± 0.05 *versus* 1.17 ± 0.18, *P* < 0.0001, 95% CI, 0.5865 to 1.636; and day 21, 0.02 ± 0.02 *versus* 1.00 ± 0.08, *P* < 0.0001, 95% CI, 0.4476 to 1.497. For two-way ANOVA, group, F (3, 95) = 42.57, *P* < 0.0001; time, F (4, 95) = 10.24, *P* < 0.0001; and interaction, F (12, 95) = 3.033, *P* = 0.0012. Two-way ANOVA revealed significant group × time interactions in all panels (*P* < 0.0001). Accordingly, *post hoc* Tukey’s tests were performed to compare SNI and sham groups at each time point. The data are presented as means ± SEM. *Red asterisks* indicate statistically significant differences between EEG sham and EEG SNI groups, whereas *black hashtags* denote statistically significant differences between non-EEG sham and non-EEG SNI groups. **, *P* < 0.01; ***, *P* < 0.001; ****, *P* < 0.0001. For EEG sham, *n* = 4 per sex; for EEG SNI, *n* = 7 per sex; for non-EEG sham, *n* = 6 per sex; and for non-EEG SNI, *n* = 6 per sex. BL, baseline; EEG, electroencephalogram; ipsi, ipsilateral.

To investigate pain development over time within each sex, male and female mice were analyzed separately. In both male and female mice, EEG and non-EEG groups that underwent nerve injury exhibited significantly reduced paw withdrawal thresholds in the ipsilateral hind paw from postsurgical day 3, compared with the sham groups (fig. 2, A and B). Dynamic allodynia scores were also significantly increased in both sexes from day 3 onward (fig. 2, C and D). Notably, no significant differences were found in the von Frey or brush test results between mice with and without telemetric transmitters, indicating that the implantation did not influence pain-like behavior. These findings confirm the development and persistence of mechanical allodynia in both male and female mice after nerve injury, independent of telemetric implantation. The progressive changes observed in behavioral testing underscore the establishment and maintenance of neuropathic pain after surgery.

### Painful Nerve Injury Reduces Rapid Eye Movement Sleep in Both Male and Female Mice

**Fig. 3.**
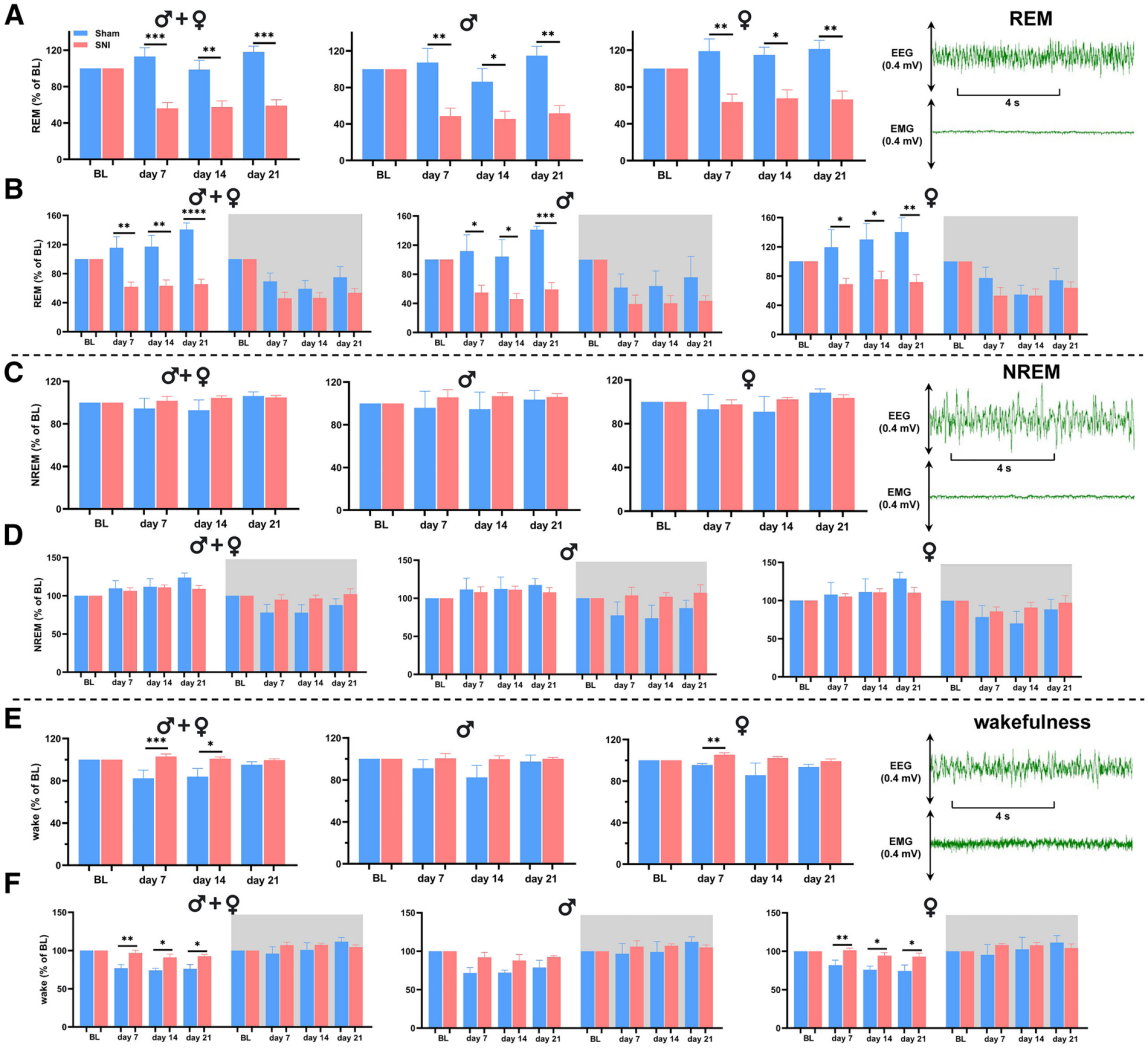
Spared nerve injury reduces REM sleep and alters wakefulness but does not affect NREM sleep duration in male and female mice. (*A*) Changes in REM sleep epochs, normalized to presurgical baseline level, were assessed on days 7, 14, and 21 after SNI. A significant reduction in REM sleep percentage was observed from day 7 and persisted through day 21 after surgery in combined data for both sexes, as well as in male and female mice separately (*left* to *right*). For sham *versus* SNI for both sexes: day 7, 113.2 ± 9.46 *versus* 56.15 ± 6.21, *P <* 0.0001, 95% CI, −80.85 to −33.19; day 14, 98.57 ± 10.15 *versus* 57.45 ± 6.83, *P* = 0.0028, 95% CI, −66.14 to −16.12; and day 21, 118.1 ± 6.37 *versus* 59.06 ± 6.33, *P <* 0.0001, 95% CI, −81.31 to −36.69. For sham *versus* SNI in males: day 7, 107.4 ± 15.38 *versus* 48.54 ± 8.70, *P* = 0.0075, 95% CI, −97.00 to −20.67; day 14, 86.29 ± 14.46 *versus* 45.43 ± 8.44, *P* = 0.0304, 95% CI, −76.75 to −4.972; and day 21, 114.8 ± 10.26 *versus* 51.62 ± 8.49, *P* = 0.0027, 95% CI, −97.24 to −29.15. For sham *versus* SNI in females: day 7, 119.0 ± 13.33 *versus* 63.76 ± 8.51, *P* = 0.0077, 95% CI, −91.27 to −19.13; day 14, 115.0 ± 8.28 *versus* 67.74 ± 9.16, *P* = 0.0151, 95% CI, −82.52 to −11.89; and day 21, 121.3 ± 9.34 *versus* 66.50 ± 9.12, *P* = 0.0027, 95% CI, −90.49 to −19.12. The *rightmost panel* shows an example of EEG/EMG signals in REM sleep. (*B*) Reduction in the amount of normalized REM sleep during the light phase was observed from day 7 onward in combined data for both sexes, as well as in male and female mice separately (*left* to *right*). For sham *versus* SNI for both sexes during light phase: day 7, 115.6 ± 15.37 *versus* 61.96 ± 6.43, *P* = 0.0012, 95% CI, −83.39 to −23.82; day 14, 117.1 ± 15.61 *versus* 63.16 ± 8.19, *P* = 0.0036, 95% CI, −87.89 to −20.05; and day 21, 140.8 ± 8.95 *versus* 65.53 ± 6.91, *P <* 0.0001, 95% CI, −100.7 to −49.78. For sham *versus* SNI in males during light phase: day 7, 111.7 ± 22.44 *versus* 54.92 ± 9.89, *P* = 0.0245, 95% CI, −104.4 to −9.180; day 14, 104.1 ± 23.49 *versus* 45.60 ± 8.15, *P* = 0.0360, 95% CI, −112.0 to −5.068; and day 21, 141.3 ± 4.58 *versus* 59.14 ± 9.54, *P* = 0.0007, 95% CI, −117.5 to −46.79. For sham *versus* SNI in females during light phase: day 7, 119.4 ± 24.26 *versus* 69.00 ± 8.06, *P* = 0.0375, 95% CI, −97.19 to −3.640; day 14, 130.1.0 ± 21.76 *versus* 75.70 ± 10.79, *P* = 0.0320, 95% CI, −103.0 to −5.832; and day 21, 140.3 ± 19.47 *versus* 71.92 ± 10.13, *P* = 0.0087, 95% CI, −114.0 to −22.70. REM sleep normalized to the baseline level during the light and dark phases when analyzed at baseline and on days 7, 14, and 21 after SNI. (*C*) NREM sleep epochs normalized to the baseline were assessed on days 7, 14 and 21 after SNI. No significant differences in NREM sleep percentage were observed in the combined data for both sexes, as well as in male and female mice separately (*left* to *right*). The *rightmost panel* shows an example of EEG/EMG signals in NREM sleep. (*D*) No significant differences in NREM sleep were observed during either light or dark phases in combined data for both sexes, as well as in male and female mice separately (*left* to *right*). (*E*) Wakefulness epochs normalized to baseline were assessed on days 7, 14, and 21 after SNI. Significant increases in wakefulness percentage were observed in combined data for both sexes during the light phase and also in female mice on day 7 (*left* to *right*). For sham *versus* SNI for both sexes: day 7, 82.30 ± 7.74 *versus* 103.10 ± 2.44, *P* = 0.0003; and day 14, 84.03 ± 7.62 *versus* 101.0 ± 1.67, *P* = 0.0240. For sham *versus* SNI in females: day 7, 95.50 ± 1.40 *versus* 105.5 ± 1.65, *P* = 0.0064, 95% CI, 3.726 to 16.37. The *rightmost panel* shows an example of EEG/EMG signals in wakefulness. (*F*) Significant increases in wakefulness were observed in combined for both sexes during the light phase and also in females (*left* to *right*). For sham *versus* SNI for both sexes during light phase: day 7, 76.75 ± 4.89 *versus* 96.76 ± 3.58, *P* = 0.0056, 95% CI, 6.658 to 33.36; day 14, 73.99 ± 2.70 *versus* 91.10 ± 4.24, *P* = 0.0326; and day 21, 76.19 ± 5.52 *versus* 92.64 ± 2.32, *P* = 0.0461. For sham *versus* SNI in females during light phase: day 7, 81.82 ± 6.73 *versus* 101.5 ± 2.57, *P* = 0.0089, 95% CI, 6.461 to 32.84; day 14, 75.84 ± 4.86 *versus* 94.29 ± 3.92, *P* = 0.0275, 95% CI, 2.632 to 34.26; and day 21, 74.31 ± 7.75 *versus* 93.04 ± 4.43, *P* = 0.0486, 95% CI, 0.1425 to 37.33. Unpaired *t* tests were performed between SNI and sham groups for each time point, with data presented as means ± SEM. For EEG sham, *n* = 4/sex; and for EEG SNI, *n* = 7/sex. *, *P* < 0.05; **, *P* < 0.01; ***, *P* < 0.001; ****, *P* < 0.0001. BL, baseline; EEG, electroencephalogram; EMG, electromyogram; NREM, non–rapid eye movement; REM, rapid eye movement; SNI, spared nerve injury.

To investigate the effects of SNI on sleep architecture, we analyzed 4-s epochs for each stage of the sleep–wake cycle at baseline (before surgery) and on postsurgical days 7, 14, and 21. REM sleep data were normalized to baseline levels for each mouse to allow for accurate comparisons. Our findings showed a significant reduction in the proportion of REM sleep on days 7, 14, and 21 after nerve injury in both male and female mice compared with sham controls (fig. 3A). To determine whether these changes were phase-dependent, we separately analyzed REM sleep during light and dark phases. The results indicated that the reduction in REM sleep occurred during the light phase in both male and female SNI mice, corresponding to the habitual resting period for mice, while no significant changes were observed during the dark phase when compared to sham controls (fig. 3B). In addition, comparison within SNI groups between day 7, 14, or 21 to the baseline level showed significant reductions in the number of REM sleep epochs for both sexes during both light and dark phase (supplemental fig. 2, A and B, https://links.lww.com/ALN/E180). Additionally, wakefulness was significantly increased in SNI, which primarily occurred during the light phase compared to sham controls (fig. 3, E and F). However, no significant differences were observed in NREM sleep across all time points after SNI (fig. 3, C and D).

### Pregabalin and Morphine Administration Reduces Mechanical Allodynia after Spared Nerve Injury

**Fig. 4.**
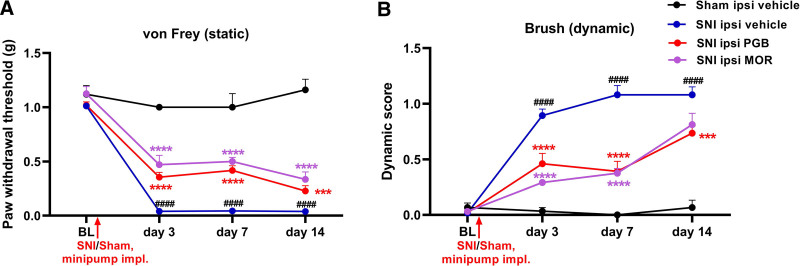
Administration of pregabalin or morphine *via* osmotic minipumps alleviates mechanical allodynia in male mice with spared nerve injury. (*A*) Reduced paw withdrawal thresholds observed in SNI + vehicle mice, compared to sham control mice, from day 3 after SNI. Administration of pregabalin or morphine led to significant increases in paw withdrawal threshold in SNI mice from day 3 after SNI. For sham vehicle *versus* SNI vehicle: day 3, 1.00 ± 0.00 g *versus* 0.04 ± 0.01 g, *P* < 0.0001, 95% CI, 0.7760 to 1.144; day 7, 1.00 ± 0.12 g *versus* 0.04 ± 0.01 g, *P* < 0.0001, 95% CI, 0.7723 to 1.139; and day 14, 1.16 ± 0.09 g *versus* 0.04 ± 0.01 g, *P* < 0.0001, 95% CI, 0.9375 to 1.304. For SNI vehicle *versus* SNI PGB: day 3, 0.04 ± 0.01 g *versus* 0.36 ± 0.04 g, *P* < 0.0001, 95% CI, −0.4339 to −0.1967; day 7, 0.04 ± 0.02 g *versus* 0.42 ± 0.04 g, *P* < 0.0001, 95% CI, −0.4909 to −0.2556; and day 14, 0.04 ± 0.01 g *versus* 0.23 ± 0.04 g, *P* < 0.001, 95% CI, −0.3061 to −0.07080. For SNI vehicle *versus* SNI MOR: day 3, 0.04 ± 0.01 g *versus* 0.47 ± 0.08 g, *P* < 0.0001, 95% CI, −0.5828 to −0.2772; day 7, 0.04 ± 0.01 g *versus* 0.50 ± 0.04 g, *P* < 0.0001, 95% CI, −0.6076 to −0.3036; and day 14, 0.04 ± 0.01g *versus* 0.33 ± 0.06 g, *P* < 0.0001, 95% CI, −0.4478 to −0.1438. For two-way ANOVA, group, F (3, 203) = 180.7, *P* < 0.0001; time, F (3, 203) = 165.1, *P* < 0.0001; and interaction, F (9, 203) = 16.62, *P* < 0.0001. (*B*) Dynamic scores were elevated in the SNI group from 3 days after SNI compared to sham mice. Administration of pregabalin or morphine resulted in a reduction of dynamic scores from 3 days after initiation of treatment. For sham vehicle *versus* SNI vehicle: day 3, 0.03 ± 0.03 *versus* 0.89 ± 0.05, *P* < 0.0001, 95% CI, −1.214 to −0.5063; day 7, 0 ± 0.00 *versus* 1.08 ± 0.08, *P* < 0.0001, 95% CI, −1.434 to −0.7263; and day 14, 0.06 ± 0.06 *versus* 1.08 ± 0.07, *P* < 0.0001, 95% CI, −1.367 to −0.6596. For SNI vehicle *versus* SNI PGB: day 3, 0.89 ± 0.05 *versus* 0.46 ± 0.09, *P* < 0.0001, 95% CI, 0.2056 to 0.6595; day 7, 1.08 ± 0.08 *versus* 0.39 ± 0.09, *P* < 0.0001, 95% CI, 0.4609 to 0.9148; and day 14, 1.08 ± 0.07 *versus* 0.73 ± 0.06, *P* < 0.001, 95% CI, 0.1177 to 0.5717. For SNI vehicle *versus* SNI MOR: day 3, 0.89 ± 0.05 *versus* 0.29 ± 0.02, *P* < 0.0001, 95% CI, 0.3084 to 0.8950; day 7, 1.08 ± 0.08 *versus* 0.37 ± 0.04, *P* < 0.0001, 95% CI, 0.4117 to 0.9983; and day 14, 1.08 ± 0.07 *versus* 0.81 ± 0.10, *P* = 0.0877, 95% CI, −0.02579 to 0.5608. For two-way ANOVA, group, F (3, 204) = 52.63, *P* < 0.0001; time, F (3, 204) = 34.55, *P* < 0.0001; and interaction, F (9, 204) = 8.103, *P* < 0.0001. Two-way ANOVA was performed with group (sham, SNI-vehicle, SNI-pregabalin, SNI-morphine) and time (days 3, 7, and 14 afterSNI) as factors. The data are presented as means ± SEM. *Black hashtags* indicate the statistically significant differences between sham vehicle and SNI vehicle group, while *red* and *purple asterisks* indicate statistically significant differences between SNI pregabalin/SNI morphine and SNI vehicle groups. For sham, *n* = 5; for SNI vehicle, *n* = 25; for SNI pregabalin, *n* = 17; and for SNI morphine, *n* = 8. ***, *P* < 0.001; ****, *P* < 0.0001. BL, baseline; ipsi, ipsilateral; MOR, morphine; PGB, pregabalin; SNI, spared nerve injury.

During the 2-week recovery period after implantation of EEG transmitters in mice, behavioral tests were conducted, confirming that sensory thresholds had returned to baseline before surgeries (supplemental fig. 3, https://links.lww.com/ALN/E181). To evaluate the effects of pregabalin and morphine on mechanical allodynia induced by neuropathic pain, we employed the SNI model, followed by implantation of osmotic minipumps for continuous delivery of vehicle, pregabalin, or morphine. Static von Frey filament and dynamic brush tests were conducted at baseline before surgery and on days 3, 7, and 14 after surgery. The results show reduced paw withdrawal thresholds in the vehicle-treated SNI mice compared with sham vehicle control mice in von Frey tests from day 3 onwards after SNI (fig. 4A, black hashtag). Additionally, scores for dynamic allodynia (brush test) were significantly increased in the SNI vehicle mice compared with sham vehicle controls (fig. 4B, black hashtag).

Pregabalin or morphine was administrated *via* intraperitoneally implanted osmotic minipumps on the same day as the SNI surgery. The results showed that the paw withdrawal threshold increased in both pregabalin-treated (fig. 4A, red asterisk) and morphine-treated (fig. 4A, purple asterisk) SNI mice, compared with vehicle-treated mice, from day 3 after injury and lasting up to the study endpoint on day 14. Furthermore, the dynamic brush test confirmed the antinociceptive effect of pregabalin and morphine in neuropathic pain mice, with a significant reduction in dynamic scores observed from day 3 to day 14 after SNI; however, no statistically significant difference was found on day 14 between morphine-treated mice and vehicle controls (fig. 4B).

### Pregabalin, but Not Morphine, Reverses REM Sleep Disturbances after Painful Nerve Injury in Mice

Next, we aimed to explore the effects of pregabalin and morphine on sleep architecture in mice with neuropathic pain. Wakefulness and sleep stages were quantified in 4-s epochs for each stage of the sleep–wake cycle per hour. The total number of epochs for wakefulness, NREM sleep, and REM sleep was analyzed over the 48-h recording period in the SNI mice treated with vehicle, pregabalin, or morphine. These analyses were conducted at baseline before injury and on days 7 and 14 after nerve injury. The epochs were normalized to the baseline for each stage in each mouse.

**Fig. 5.**
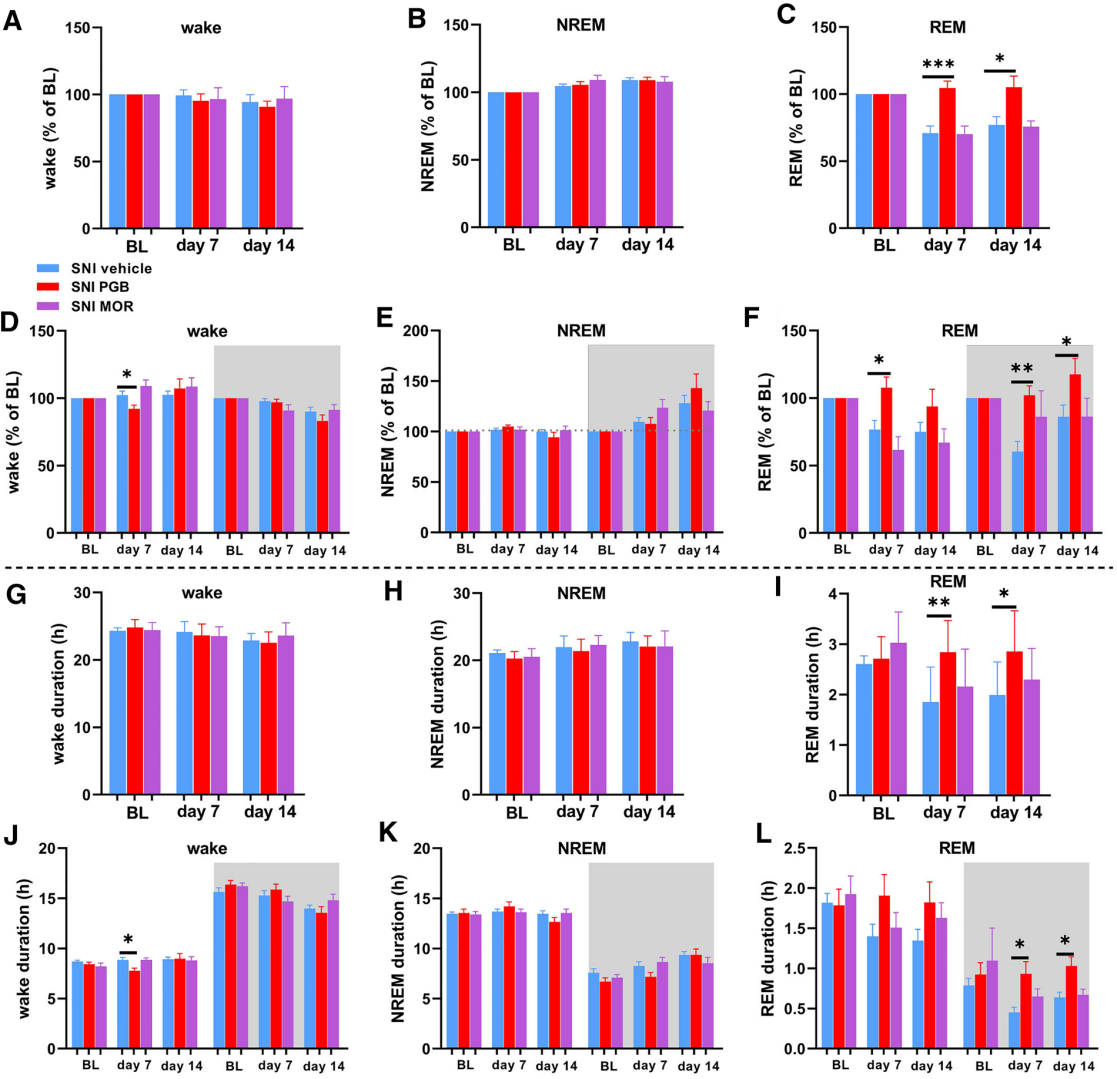
Continuous administration of pregabalin restores the REM sleep to presurgical levels after spared nerve injury. EEG recordings were performed at baseline before SNI and on days 7 and 14 after SNI for 48 h at each time point, with data analyzed in 4-s epochs for each stage of the sleep–wake cycle. (*A–F*) Total number of epochs for each sleep–wake stage normalized to baseline for each mouse. (*A*) No significant differences in wakefulness were observed in pregabalin- or morphine-treated SNI mice, compared to vehicle-treated SNI mice. (*B*) Pregabalin or morphine had no significant effect on NREM sleep in the SNI mice. (*C*) REM sleep epochs significantly increased on days 7 and 14 after SNI in pregabalin-treated mice, while no significant differences were observed in morphine-treated mice, compared to vehicle-treated SNI mice. For SNI vehicle *versus* SNI PGB: day 7, 70.8 ± 5.37 *versus* 104.6 ± 5.08, *P* = 0.0008, 95% CI, 16.08 to 51.47; and day 14, 77.08 ± 6.01 *versus* 105.1 ± 8.38, *P* = 0.0142, 95% CI, 6.294 to 49.71. (*D*) When wakefulness epochs were analyzed separately for light and dark phases, significant reductions in wakefulness were observed in the pregabalin-treated SNI mice during the light phase on day 7 after SNI, compared to vehicle-treated SNI mice. For SNI vehicle *versus* SNI PGB during the light phase: day 7, 102.3 ± 3.03 *versus* 92.14 ± 2.69, *P* = 0.0454, 95% CI, −20.02 to −0.2291. (*E*) No significant differences in the NREM sleep were observed in either pregabalin- or morphine-treated SNI mice. (*F*) REM sleep epochs were significantly increased in pregabalin-treated SNI mice on day 7 during the light phase and on both days 7 and 14 during the dark phase, while no significant differences were observed in morphine-treated SNI mice during either phase, compared to vehicle-treated SNI mice. For SNI vehicle *versus* SNI PGB during the light phase: day 7, 76.63 ± 6.70 *versus* 107.6 ± 7.84, *P* = 0.0111, 95% CI, 7.927 to 54.03. For SNI vehicle *versus* SNI PGB during dark phase: day 7, 60.43 ± 7.45 *versus* 102.1 ± 6.99, *P* = 0.0021, 95% CI, 17.14 to 66.16; and day 14, 86.23 ± 8.51 *versus* 117.6 ± 11.68, *P* = 0.0450, 95% CI, 0.7807 to 61.96. (*G–L*) Total duration (in hours) for each sleep–wake stage across 48 h of the recording. (*G*, *H*) No significant differences were observed in wakefulness duration (*G*) or NREM sleep duration (*H*) across treatment groups. (*I*) REM sleep duration was significantly increased in pregabalin-treated mice on both days 7 and day 14 after SNI. For SNI vehicle *versus* SNI PGB: day 7, 1.853 ± 0.18 h *versus* 2.839 ± 0.23 h, *P* = 0.0051, 95% CI, 0.3341 to 1.638; and day 14, 1.986 ± 0.17 h *versus* 2.855 ± 0.30 h, *P* = 0.0161, 95% CI, 0.1805 to 1.558. (*J*) Wakefulness duration was reduced in pregabalin-treated mice during the light phase on day 7. For SNI vehicle *versus* SNI PGB during light phase: day 7, 8.860 ± 0.24 h *versus* 7.759 ± 0.27 h, *P* = 0.0128, 95% CI, −1.939 to −0.2626. (*K*) No significant differences were observed in NREM sleep duration. (*L*) REM sleep duration was significantly increased in pregabalin-treated mice during the dark phase on both days 7 and 14, with no significant differences observed during the light phase. For SNI vehicle *versus* SNI PGB during dark phase: day 7, 0.4521 ± 0.06 h *versus* 0.9335 ± 0.15 h, *P* = 0.0011; and day 14, 0.6377 ± 0.06 h *versus* 1.033 ± 0.11 h, *P* = 0.0035, 95% CI, 0.1472 to 0.6431. Morphine had no significant effect on sleep duration at any time point compared to vehicle-treated SNI mice. Unpaired *t* tests were performed between the SNI PGB and SNI vehicle groups, as well as between the SNI MOR and SNI vehicle groups, for each time point. The data are presented as means ± SEM. For SNI vehicle, *n* = 15; for SNI PGB, *n* = 7; and for SNI MOR, *n* = 8. *, *P* < 0.05; **, *P* < 0.01. BL, baseline; EEG, electroencephalogram; MOR, morphine; PGB, pregabalin; REM, rapid eye movement; SNI, spared nerve injury.

Compared with vehicle treatment, REM sleep epochs were significantly increased in the pregabalin-treated mice on both days 7 and 14 after surgery, while morphine administration had no significant effect (fig. 5C). Consistently, the number of REM sleep epochs was also significantly increased in pregabalin-treated mice on both days 7 and 14 compared with vehicle-treated mice (supplemental fig. 4C, https://links.lww.com/ALN/E182). In contrast, within-group comparisons showed that REM sleep epochs were significantly reduced in vehicle- and morphine-treated SNI mice on days 7 and 14 relative to their respective baseline levels (supplemental fig. 4C, https://links.lww.com/ALN/E182). However, no significant differences were observed in pregabalin-treated mice at either time point compared to baseline (supplemental fig. 4C, https://links.lww.com/ALN/E182). Neither pregabalin nor morphine significantly affected the epochs for wakefulness (fig. 5A; supplemental fig. 4A, https://links.lww.com/ALN/E182) or NREM sleep (fig. 5B; supplemental fig. 4B, https://links.lww.com/ALN/E182).

Given that mice are nocturnal animals and sleep during the light phase, we further analyzed sleep–wake stages during the light and dark phases separately. A significant reduction in wakefulness during the light phase was observed in the pregabalin-treated SNI mice compared with the vehicle group on day 7 after injury (fig. 5D). Consistently, the number of wakefulness epochs was also significantly reduced on day 7 in morphine-treated SNI mice during the light phase compared to vehicle-treated controls (supplemental fig. 4D, https://links.lww.com/ALN/E182). No significant differences in NREM sleep were observed for either the pregabalin- or morphine-treated mice, compared with SNI vehicle mice (fig. 5E; supplemental fig. 4E, https://links.lww.com/ALN/E182). However, within-group comparisons revealed an increase in NREM sleep during the dark phase in both the SNI vehicle and SNI morphine groups on day 7 compared with their respective baseline levels. Additionally, all three groups exhibited increased NREM sleep on day 14 relative to their own baseline levels (fig. 5E). Numbers of REM sleep epochs were increased in the pregabalin-treated mice on day 7 during the light phase and on both days 7 and 14 during the dark phase. In contrast, morphine-treated mice showed no significant difference in REM sleep compared with the vehicle-treated mice (fig. 5F). Similarly, analysis of REM sleep epochs confirmed a significant increase in pregabalin-treated mice compared with vehicle-treated controls (supplemental fig. 4F, https://links.lww.com/ALN/E182). In contrast, within-group comparisons showed that REM sleep was significantly reduced in morphine-treated SNI mice on both days 7 and 14 relative to baseline levels (supplemental fig. 4F, https://links.lww.com/ALN/E182).

To complement the analysis of sleep–wake stage distribution based on the number of epochs, we further quantified the total duration of each sleep–wake stage in hours across the 48-h recording periods. Consistent with findings from the epoch data, no significant differences were observed in durations of wakefulness (fig. 5G) or NREM sleep (fig. 5H) across treatment groups. However, REM sleep duration was significantly increased in the pregabalin-treated mice compared to the vehicle group on both days 7 and 14 after SNI (fig. 5I).

In the analysis of light and dark phases, wakefulness duration was reduced in pregabalin-treated SNI mice during the light phase on day 7 (fig. 5J), while no significant differences were found for NREM sleep duration (fig. 5K). Regarding REM sleep duration, a significant increase was observed in pregabalin-treated mice during the dark phase on both days 7 and 14, with no significant differences during the light phase (fig. 5L). Morphine treatment did not affect the duration of sleep stages. These findings suggest that pregabalin may selectively restore REM sleep continuity during the active (dark) phase in mice with neuropathic pain.

### Pregabalin and Morphine Exhibit Distinct Effects on Sleep Spindles during Transitions between Wakefulness, NREM Sleep, and REM Sleep

We investigated the effect of pregabalin and morphine on sleep spindles, which occur during NREM sleep and influence sensory and affective processing and memory consolidation.^52^ We quantified the total number of spindles and their average duration over a 24-h recording period, encompassing both light and dark phases. Spindle activity was further analyzed during NREM sleep and transitions between NREM and REM sleep, as well as between wakefulness and NREM sleep.

**Fig. 6.**
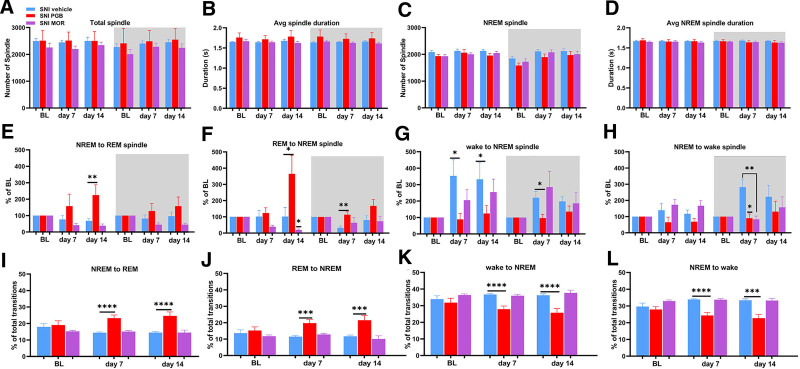
Sleep spindle counts are increased during NREM and REM sleep transitions in pregabalin-treated mice. (*A*) Total spindle counts during light and dark phases at BL, day 7, and day 14 after SNI showed no significant differences between pregabalin- or morphine-treated groups and vehicle-treated controls. (*B*) Average spindle duration remained unchanged across all treatment groups during light and dark phases at BL, day 7, and day 14 after SNI. (*C*) Spindle counts during NREM sleep showed no significant differences among the treatment groups. (*D*) Average spindle duration during NREM sleep was not significantly affected by pregabalin or morphine administration. (*E*) Pregabalin-treated mice demonstrated increased spindle counts during the transition from NREM to REM sleep in the light phase on day 14 after SNI, whereas no changes were observed in the morphine-treated group. For SNI vehicle *versus* SNI PGB during the light phase: day 14, 67.96 ± 12.92 *versus* 224.9 ± 62.89, *P* = 0.0045, 95% CI, 55.17 to 258.7. (*F*) Spindle counts during the transition from REM to NREM sleep increased in pregabalin-treated mice during the light phase on day 14 and the dark phase on day 7 after SNI. For SNI vehicle *versus* SNI PGB during the light phase: day 14, 101.8 ± 55.71 *versus* 365.2 ± 113.4, *P* = 0.0416; SNI vehicle *versus* SNI PGB during the dark phase: and day 7, 32.72 ± 11.46 *versus* 113.8 ± 16.83, *P* = 0.0022. Morphine-treated mice showed decreased spindle counts during the light phase on day 14 after SNI. For SNI vehicle *versus* SNI MOR during the light phase: day 14, 101.8 ± 55.71 *versus* 18.49 ± 6.47, *P* = 0.0456. (*G*) Pregabalin-treated mice exhibited reduced spindle counts during the transition from wakefulness to NREM sleep in the light phase on days 7 and 14 and in the dark phase on day 7 after SNI. For SNI vehicle *versus* SNI PGB during the light phase: day 7, 353.3 ± 98.38 *versus* 88.81 ± 35.57, *P* = 0.0106; day 14, 332.5 ± 101.9 *versus* 124.5 ± 48.10, *P* = 0.0263; SNI vehicle *versus* SNI PGB during the dark phase: and day 7, 220.9 ± 37.07 *versus* 97.27 ± 22.33, *P* = 0.0375, 95% CI, −239.4 to −7.946. No significant changes were observed in the morphine-treated group. (*H*) During the transition from NREM sleep to wakefulness, spindle counts decreased in pregabalin-treated mice during both the light and dark phases on day 7 after SNI. For SNI vehicle *versus* SNI PGB during the dark phase: day 7, 282.2 ± 49.69 *versus* 90.36 ± 25.90, *P* = 0.0147. Morphine-treated mice exhibited reductions only in the dark phase on day 7 after SNI. For SNI vehicle *versus* SNI MOR during the dark phase: day 7, 282.2 ± 49.69 *versus* 83.04 ± 20.31, *P* = 0.0057, 95% CI, −332.7 to −65.65. (*I*) The proportion of NREM-to-REM sleep transitions significantly increased in pregabalin-treated mice compared to vehicle-treated mice on both days 7 and 14 after SNI. For SNI vehicle *versus* SNI PGB: day 7, 14.40 ± 0.41 *versus* 23.26 ± 1.88, *P* < 0.0001, 95% CI, 5.866 to 11.86; and day 14, 14.51 ± 0.62 *versus* 24.60 ± 2.43, *P* < 0.0001, 95% CI, 6.114 to 14.06. No significant differences were observed in the morphine-treated group. (*J*) The proportion of REM-to-NREM sleep transitions significantly increased in pregabalin-treated mice compared to vehicle-treated mice on both days 7 and 14 after SNI. For (SNI vehicle *versus* SNI PGB: day 7, 11.52 ± 0.69 *versus* 19.79 ± 2.10, *P* = 0.0002, 95% CI, 4.601 to 11.94; and day 14, 11.76 ± 0.75 *versus* 21.54 ± 2.87, *P* = 0.0004, 95% CI, 5.069 to 14.50. No significant changes were observed in the morphine-treated group. (*K*) Pregabalin-treated mice showed a significant reduction in the proportion of transitions from wakefulness to NREM sleep compared to vehicle-treated controls on days 7 and 14 after SNI. For SNI vehicle *versus* SNI PGB: day 7, 36.74 ± 0.57 *versus* 27.87 ± 1.89, *P* < 0.0001, 95% CI, −12.10 to −5.637; and day 14, 36.27 ± 0.88 *versus* 25.72 ± 2.52, *P* < 0.0001, 95% CI, −15.05 to −6.053. No significant differences were observed in the morphine-treated group. (*L*) The proportion of transitions from NREM sleep to wakefulness decreased in pregabalin-treated mice compared to vehicle-treated controls on days 7 and 14 after SNI. For SNI vehicle *versus* SNI PGB: day 7, 33.87 ± 0.37 *versus* 24.33 ± 1.70, *P* < 0.0001, 95% CI, −12.24 to −6.838; and day 14, 34.43 ± 0.86 *versus* 22.77 ± 2.15, *P* = 0.0001. Morphine-treated mice showed no significant changes. Unpaired *t* tests were performed between the SNI PGB and SNI vehicle groups, as well as between the SNI MOR and SNI vehicle groups, for each time point. The data are presented as means ± SEM. For SNI vehicle, *n* = 15; for SNI PGB, *n* = 7; and for SNI MOR, *n* = 8. *, *P* < 0.05; **, *P* < 0.01. Avg, average; BL, baseline; MOR, morphine; NREM, non–rapid eye movement; PGB, pregabalin; REM, rapid eye movement; SNI, spared nerve injury.

Our analysis revealed no statistically significant differences in the total number or average duration of spindles among SNI mice treated with vehicle, pregabalin, or morphine (fig. 6, A and B). Similarly, there were no significant differences in spindle counts or durations during NREM sleep between the treatment groups (fig. 6, C and D). However, significant effects of pregabalin were observed during specific transition phases. During the transition from NREM to REM sleep, spindle counts increased significantly in the pregabalin-treated mice during the light phase on day 7 after SNI, compared with the vehicle controls, whereas no significant changes were observed in the morphine-treated group (fig. 6E; supplemental fig. 5A, https://links.lww.com/ALN/E183). For the transition from REM to NREM sleep, pregabalin-treated mice showed increased spindle counts during the light phase on day 14 after injury and during the dark phase on day 7. In contrast, spindle counts decreased in the morphine-treated group during the light phase on day 14 (fig. 6F; supplemental fig. 5B, https://links.lww.com/ALN/E183).

During the transition from wakefulness to NREM sleep, spindle counts were reduced in pregabalin-treated mice during the light phase on days 7 and 14 after SNI, as well as during the dark phase on day 7. No significant differences were observed in the morphine-treated group during this transition (fig. 6G). Finally, during the transition from NREM sleep to wakefulness, spindle counts decreased in the pregabalin-treated mice during and dark phase on day 7 after SNI, and morphine-treated mice exhibited significant reductions during the dark phase on day 7 (fig. 6H).

To assess the contribution of different sleep–wake transitions, we calculated the relative proportion of transitions between NREM and REM sleep, as well as between wakefulness and NREM sleep, out of all transition events.^46^ The proportion of NREM to REM transitions was significantly increased in pregabalin-treated SNI mice compared to vehicle-treated mice on both days 7 and 14 after SNI surgery, while no significant differences were observed in morphine-treated mice compared to vehicle controls (fig. 6, I and, J). Conversely, the proportion between wake-to-NREM transitions was significantly decreased in pregabalin-treated SNI mice on days 7 and 14 after SNI compared to vehicle-treated mice, with no significant changes observed in morphine-treated mice (fig. 6, K and L).

### Pregabalin Alters REM Sleep Power Spectrum in Spared Nerve Injury Mice, While Morphine Shows No Significant Effect

To assess the effects of pregabalin and morphine on EEG power across vigilance states, we conducted power spectral analysis across both the 0.5- to 34.5-Hz frequency and the 30- to 50-Hz low gamma range, using 0.5-Hz bins during a 24-h period, encompassing both light and dark phases.

**Fig. 7.**
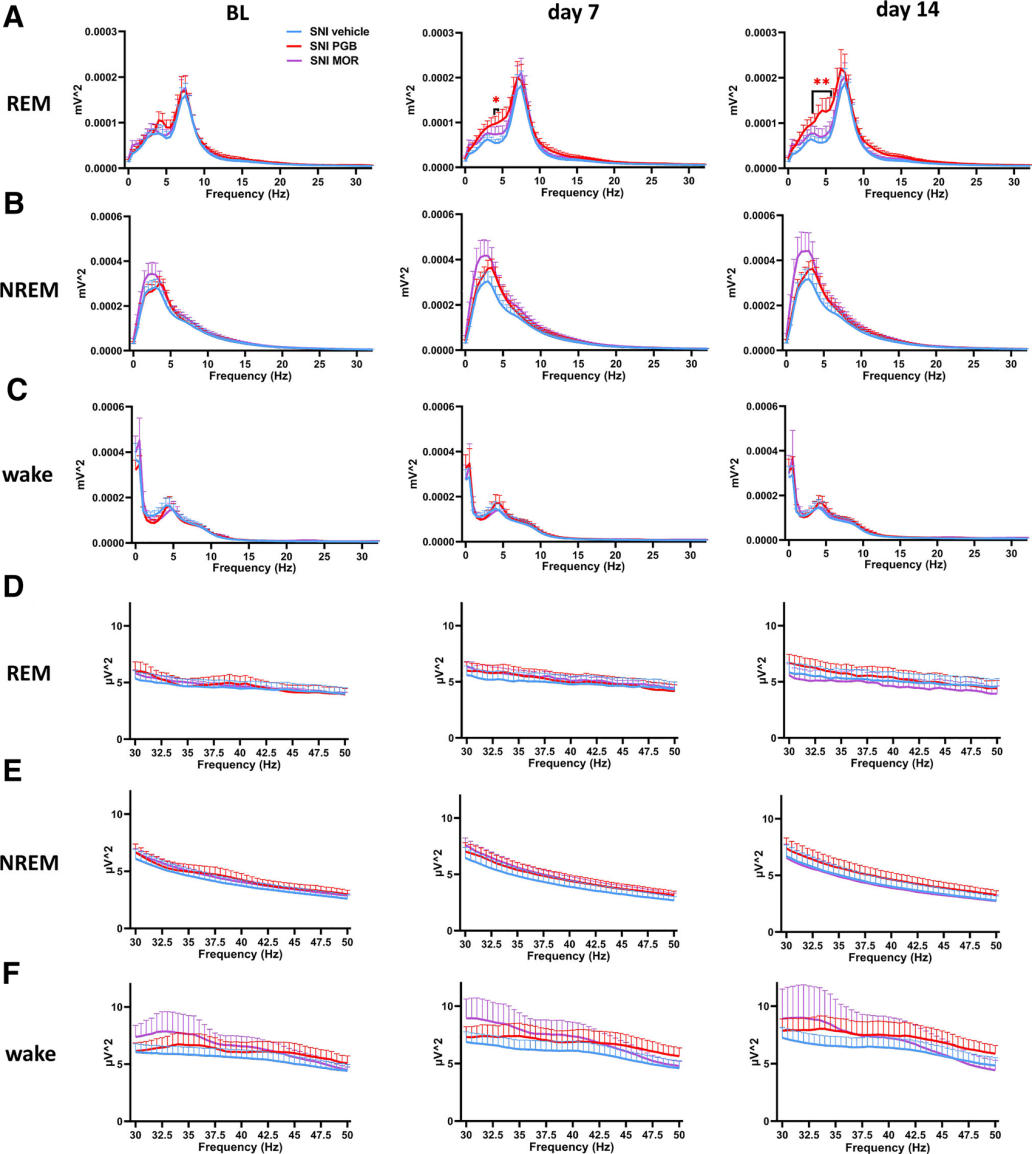
Alterations in EEG power spectra during the light phase in SNI mice treated with pregabalin or morphine. (*A*) REM sleep power spectra at BL, day 7, and day 14 after SNI. Pregabalin-treated mice exhibited significantly increased power in the 4- to 4.5-Hz range on day 7), which extended to the 3.5- to 5.5-Hz range by day 14, compared with SNI vehicle mice For SNI vehicle *versus* SNI PGB during light phase: 4 Hz, 5.39 × 10^−5^ ± 8.15 × 10^−6^ mV^2^ *versus* 9.69 × 10^−5^ ± 1.71 × 10^−5^ mV^2^, *P* = 0.0211, 95% CI, 7.28 × 10^−6^ to 7.87 × 10^−5^; and 4.5 Hz, 5.44 × 10^−5^ ± 8.80 × 10^−6^ mV^2^ *versus* 1.01 × 10^−4^ ± 2.16 × 10^−5^ mV^2^, *P* = 0.0286, 95% CI, 5.44 × 10^−6^ to 8.69 × 10^−5^. For SNI vehicle *versus* SNI PGB during light phase: 3.5 Hz, 6.05 × 10^−5^ ± 1.02 × 10^−5^ mV^2^ *versus* 1.04 × 10^−4^ ± 1.69 × 10^−5^ mV^2^, *P* = 0.0431, 95% CI, 1.51 × 10^−6^ to 8.49 × 10^−5^; 4 Hz, 5.69 × 10^−5^ ± 1.04 × 10^−5^ mV^2^ *versus* 1.20 × 10^−4^ ± 2.00 × 10^−5^ mV^2^, *P* = 0.0085, 95% CI, 1.82 × 10^−5^ to 1.07 × 10^−4^; 4.5 Hz, 5.64 × 10^−5^ ± 1.08 × 10^−5^ mV^2^ *versus* 1.28 × 10^−4^ ± 2.64 × 10^−5^ mV^2^, *P* = 0.0080, 95% CI, 2.11 × 10^−5^ to 1.21 × 10^−4^; 5 Hz, 5.89 × 10^−5^ ± 1.04 × 10^−5^ mV^2^ *versus* 1.25 × 10^−4^ ± 2.95 × 10^−5^ mV^2^, *P* = 0.0147, 95% CI, 1.47 × 10^−5^ to 1.17 × 10^−4^; and 5.5 Hz, 6.90 × 10^−5^ ± 1.22 × 10^−5^ mV^2^ *versus* 1.29 × 10^−4^ ± 2.69 × 10^−5^ mV^2^, *P* = 0.0313, 95% CI, 6.10 × 10^−6^ to 1.14 × 10^−4^. (*B*) NREM sleep power spectra showed no significant differences among treatment groups. (*C*) Wakefulness power spectra also showed no significant differences between SNI vehicle and either SNI pregabalin or SNI morphine mice. (*D–F*) Low gamma (30 to 50 Hz) power spectra during the light phase. No significant differences were observed in REM (*D*), NREM (*E*), or wakefulness (*F*) states among the treatment groups. *Red* and *purple asterisks* indicate statistically significant differences between SNI PGB/SNI MOR and SNI vehicle groups, respectively. Unpaired *t* tests were performed between the SNI PGB and SNI vehicle groups, as well as between the SNI MOR and SNI vehicle groups, for each time point. The data are presented as means ± SEM. For SNI vehicle, *n* = 15; for SNI PGB, *n* = 7; and for SNI MOR, *n* = 8. *, *P* < 0.05; **, *P* < 0.01. BL, baseline; EEG, electroencephalogram; MOR, morphine; NREM, non–rapid eye movement; PGB, pregabalin; REM, rapid eye movement; SNI, spared nerve injury.

**Fig. 8.**
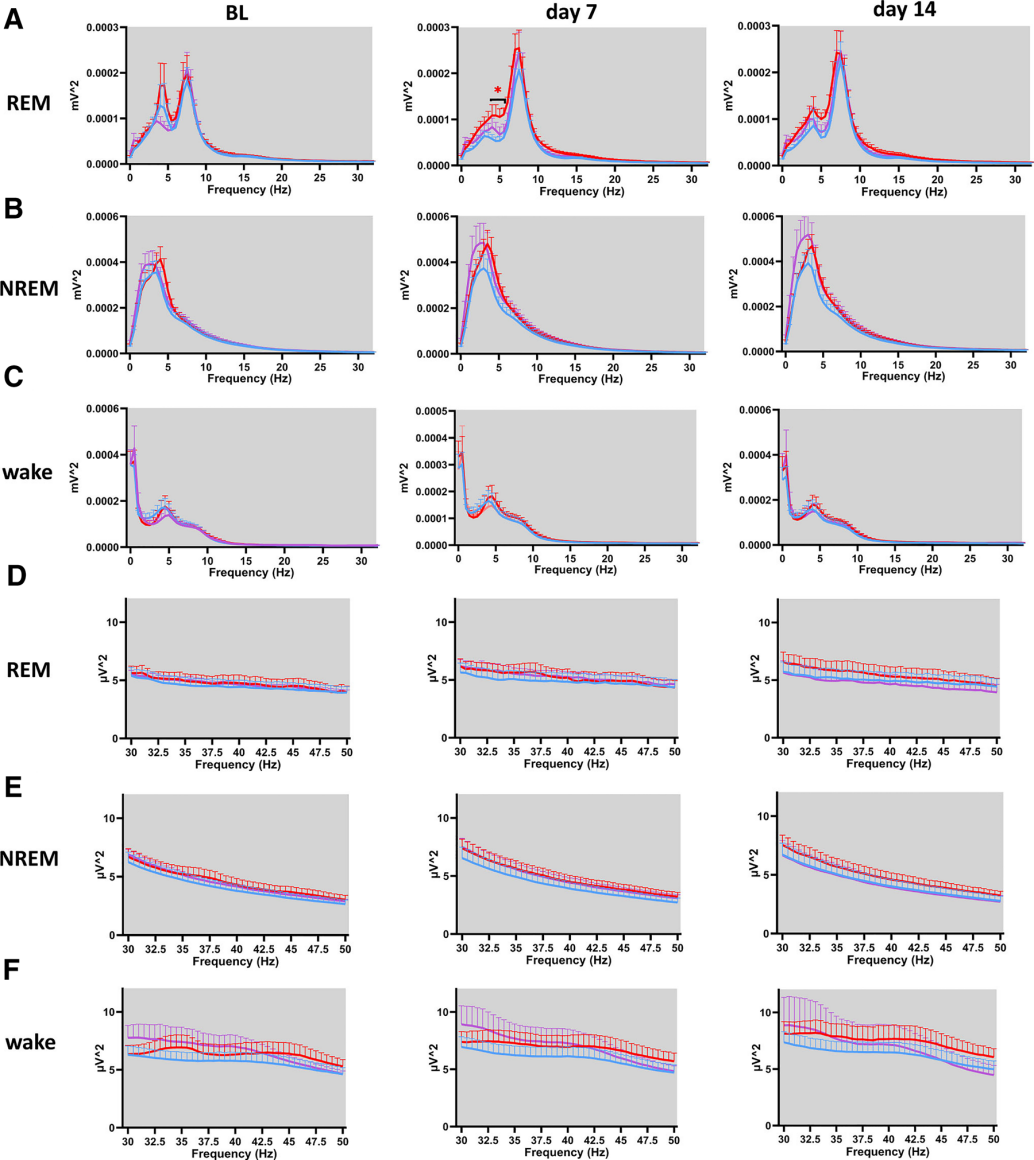
Alterations in EEG power spectra during the dark phase in SNI mice treated with pregabalin or morphine. (*A*) REM sleep power spectra at BL, day 7, and day 14 after SNI. On day 7, pregabalin-treated mice showed increased power in the 4- to 5.5-Hz range compared with SNI vehicle mice. For SNI vehicle *versus* SNI PGB during dark phase: 4 Hz, 5.77 × 10^−5^ ± 9.50 × 10^−6^ mV^2^ *versus* 1.09 × 10^−4^ ± 2.34 × 10^−5^ mV^2^, *P* = 0.0310, 95% CI, 5.28 × 10^−6^ to 9.64 × 10^−5^; 4.5 Hz, 5.31 × 10^−5^ ± 8.87 × 10^−6^ mV^2^ *versus* 1.09 × 10^−4^ ± 2.34 × 10^−5^ mV^2^, *P* = 0.0155, 95% CI, 1.20 × 10^−5^ to 9.88 × 10^−5^; 5 Hz, 5.38 × 10^−5^ ± 9.72 × 10^−6^ mV^2^ *versus* 1.05 × 10^−4^ ± 1.77 × 10^−5^ mV^2^, *P* = 0.0233, 95% CI, 7.97 × 10^−6^ to 9.49 × 10^−5^; and 5.5 Hz, 6.03 × 10^−5^ ± 1.07 × 10^−5^ mV^2^ *versus* 1.11 × 10^−4^ ± 1.40 × 10^−5^ mV^2^, *P* = 0.329, 95% CI, 4.63 × 10^−6^ to 9.62 × 10^−5^. No significant changes were observed for morphine-treated mice. (*B*) NREM sleep power spectra showed no significant differences among groups. (*C*) Wakefulness power spectra also did not differ significantly between treatment groups. (*D–F*) Low gamma (30 to 50 Hz) power spectra during the dark phase. No significant differences were observed in REM (*D*), NREM (*E*), or wakefulness (*F*) states among the treatment groups. *Red* and *purple asterisks* indicate statistically significant differences between SNI PGB/SNI MOR and SNI vehicle groups, respectively. Unpaired *t* tests were performed between the SNI PGB and SNI vehicle groups, as well as between the SNI MOR and SNI vehicle groups, for each time point. The data are presented as means ± SEM. For SNI vehicle, n = 15; for SNI PGB, n = 7; and for SNI MOR, n = 8. *, *P* < 0.05; **, *P* < 0.01. BL, baseline; EEG, electroencephalogram; MOR, morphine; NREM, non–rapid eye movement; PGB, pregabalin; REM, rapid eye movement; SNI, spared nerve injury.

In the pregabalin-treated SNI mice, significant increases in REM sleep power spectra were observed compared to vehicle-treated SNI mice. On day 7 after injury, pregabalin-treated mice exhibited elevated EEG power in the 4- to 4.5-Hz range during the light phase (fig. 7A, middle). By day 14 after injury, this increase extended to the 3.5- to 5.5-Hz range during the light phase (fig. 7A, right). However, no significant differences in low gamma power were detected between SNI vehicle and either pregabalin- or morphine-treated SNI mice during the light phase (fig. 7, D to F). During the dark phase, similar enhancement in REM power was observed: pregabalin-treated mice showed increased power within the 4- to 5.5-Hz range on day 7 after SNI (fig. 8A, middle). Conversely, morphine-treated mice did not show significant changes in REM sleep power spectra during either light or dark phases (figs. 7A and 8A). Additionally, no significant differences in power spectra were observed among the treatment groups during NREM sleep (fig. 7B and 8B) or wakefulness (fig. 7C and 8C) in either the light or dark phases. Consistent with the findings during the light phase, there were no significant differences in low gamma (30 to 50 Hz) power during the dark phase between SNI vehicle and either pregabalin- or morphine-treated groups (fig. 8, D to F).

### Pregabalin Restores Circadian Rhythmicity of Locomotor Activity and Body Temperature More Effectively than Morphine after Nerve Injury

To investigate the effects of pregabalin and morphine on circadian rhythmicity in association with antinociception, we analyzed variations in circadian parameters, including mesor, amplitude, and acrophase, for both locomotor activity and body temperature.

**Fig. 9.**
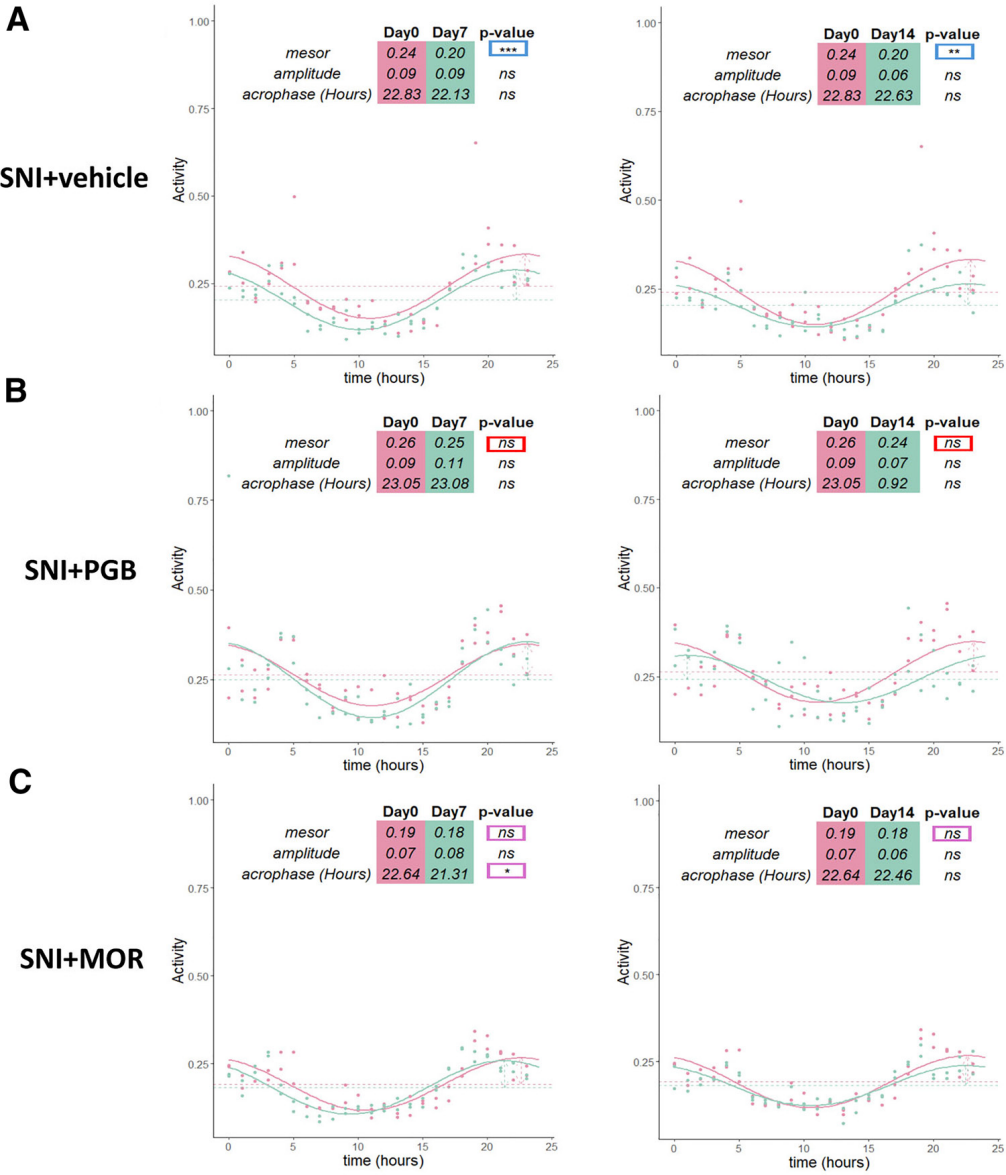
Impact of pregabalin and morphine on circadian rhythmicity of locomotor activity in spared nerve injury mice. Cosinor analysis was performed for locomotor activity of mice over a 24-h period at days 7 and 14 after SNI, compared to presurgical baseline level (day 0). (*A*) Circadian rhythmicity parameters in vehicle-treated SNI mice on days 7 and 14 after SNI. A significant disruption in the mesor was observed at both time points. For SNI vehicle, day 0 (BL) *versus* day 7: mesor estimate, 0.2414 *versus* 0.2027, *P* = 0.0040; and day 0 (BL) *versus* day 14: mesor estimate, 0.2414 *versus* 0.2039, *P* = 0.0070. (*B*) Pregabalin restored the mesor parameter in locomotor activity on days 7 and 14 after SNI, indicating improved circadian rhythmicity. For SNI PGB, day 0 (BL) *versus* day 7: mesor estimate, 0.2631 *versus* 0.2501, *P* = 0.4432; and day 0 (BL) *versus* day 14: mesor estimate, 0.2631 *versus* 0.2430, *P* = 0.1566. (*C*) Morphine improved the mesor parameter on days 7 and 14 after SNI but caused a disturbance in the acrophase parameter on day 7. For SNI MOR, day 0 (BL) *versus* day 7: mesor estimate, 0.1910 *versus* 0.1818, *P* = 0.2432; acrophase estimate, 22.6366 *versus* 21.3125, *P* = 0.0208; and day 0 (BL) *versus* day 14: mesor estimate, 0.1910 *versus* 0.1798, *P* = 0.1549. *Blue rectangles* indicate circadian parameters that show statistically significant differences between baseline (day 0) and postsurgical days 7 or 14 in the SNI vehicle group. *Red rectangles* highlight the reversal of these differences in the SNI PGB group. *Purple rectangles* indicate the reversal of the differences observed in the SNI vehicle group, as well as additional significant differences uniquely induced in the SNI MOR group on day 7. For SNI vehicle, *n* = 15; for SNI PGB, *n* = 7; and for SNI MOR, *n* = 8. *, *P* < 0.05; **, *P* < 0.01; ***, *P* < 0.001. BL, baseline; MOR, morphine; PGB, pregabalin; SNI, spared nerve injury.

Locomotor activity was exclusively associated with wakefulness in our recordings, as activity values were zero during both NREM and REM sleep. Consequently, rhythmicity analyses of locomotor activity were limited to wakefulness periods. Our findings revealed that the mesor parameter for locomotor activity was significantly disrupted in the vehicle-treated SNI mice on both days 7 and 14 after injury, compared to baseline before nerve injury (fig. 9A). Pregabalin treatment effectively restored the mesor disturbance induced by SNI on both days (fig. 9B). Morphine administration also restored the mesor parameter on both days but caused a disruption in the acrophase on day 7 (fig. 9C).

**Fig. 10.**
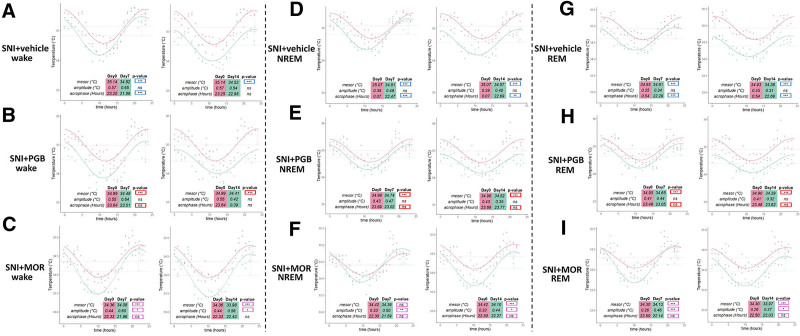
Vigilance state-specific effects of pregabalin and morphine on body temperature rhythms in spared nerve injury mice. Cosinor analysis was conducted separately for 24-h body temperature data of mice during wakefulness (*A–C*), NREM sleep (*D–F*), and REM sleep (*G–I*) on days 7 and 14 after SNI, compared with presurgical baseline level (day 0). (*A*, *D*, *G*) Vehicle-treated SNI mice showed disrupted mesor and acrophase parameters across all vigilance states, particularly on day 7. For wakefulness day 0 (BL) *versus* day 7: mesor estimate, 35.1362 *versus* 34.8172, *P* < 0.0001; acrophase estimate, 23.2459 *versus* 21.9867, *P* = 0.0030; wakefulness day 0 (BL) *versus* day 14: mesor estimate, 35.1362 *versus* 34.5339, *P* < 0.0001; NREM day 0 (BL) *versus* day 7: mesor estimate, 35.0670 *versus* 34.8112, *P* < 0.0001; acrophase estimate, 0.0724 *versus* 22.4696, *P* = 0.0004; NREM day 0 (BL) *versus* day 14: mesor estimate, 35.0670 *versus* 34.5659, *P* < 0.0001; acrophase estimate, 0.0724 *versus* 22.6898, *P* = 0.0109; REM day 0 (BL) *versus* day 7: mesor estimate, 34.9257 *versus* 34.6128, *P* < 0.0001; acrophase estimate, 0.5432 *versus* 22.2910, *P* < 0.0001; and REM day 0 (BL) *versus* day 14: mesor estimate, 34.9257 *versus* 34.3819, *P* < 0.0001; acrophase estimate, 0.5432 *versus* 22.6945, *P* = 0.0015. (*B*, *E*, *H*) Pregabalin treatment significantly restored the acrophase in all vigilance states, but mesor impairments remained. For wakefulness day 0 (BL) *versus* day 7: mesor estimate, 34.9857 *versus* 34.4790, *P* < 0.0001; acrophase estimate, 23.6416 *versus* 23.0114, *P* = 0.3371; wakefulness day 0 (BL) *versus* day 14: mesor estimate, 34.9857 *versus* 34.4067, *P* = 0.2395; NREM day 0 (BL) *versus* day 7: mesor estimate, 34.9829 *versus* 34.7415, *P* < 0.0001; acrophase estimate, 23.6901 *versus* 23.0217, *P* = 0.2943; NREM day 0 (BL) *versus* day 14: mesor estimate, 34.9829 *versus* 34.5164, *P* < 0.0001; acrophase estimate, 23.6901 *versus* 23.7657, *P* = 0.9287; REM day 0 (BL) *versus* day 7: mesor estimate, 34.9035 *versus* 34.6490, *P* < 0.0001; acrophase estimate, 23.4822 *versus* 23.0549, *P* = 0.5240; and REM day 0 (BL) *versus* day 14: mesor estimate, 34.9035 *versus* 34.3887, *P* < 0.0001; acrophase estimate, 23.4823 *versus* 23.6228, *P* = 0.8647. (*C*, *F*, *I*) Morphine restored acrophase on both days 7 and 14 but induced consistent amplitude disruptions across all states and postsurgical time points. For wakefulness day 0 (BL) *versus* day 7: mesor estimate, 34.3572 *versus* 34.0844, *P* < 0.0001; acrophase estimate, 22.3346 *versus* 21.8613, *P* = 0.3632; amplitude estimate, 0.4384 *versus* 0.6042, *P* = 0.0168; wakefulness day 0 (BL) *versus* day 14: mesor estimate, 34.3572 *versus* 33.9845, *P* < 0.0001; acrophase estimate, 22.3346 *versus* 22.4306, *P* = 0.8570; amplitude estimate, 0.4384 *versus* 0.5793, *P* = 0.0433; NREM day 0 (BL) *versus* day 7: mesor estimate, 34.4203 *versus* 34.3912, *P* = 0.5003; acrophase estimate, 22.3006 *versus* 21.5918, *P* = 0.2412; amplitude estimate, 0.3259 *versus* 0.5004, *P* = 0.0050; NREM day 0 (BL) *versus* day 14: mesor estimate, 34.4203 *versus* 34.1010, *P* < 0.0001; acrophase estimate, 22.3006 *versus* 22.2746, *P* = 0.9619; amplitude estimate, 0.3259 *versus* 0.4403, *P* = 0.0330; REM day 0 (BL) *versus* day 7: mesor estimate, 34.2977 *versus* 34.1334, *P* < 0.0001; acrophase estimate, 22.5019 *versus* 22.1436, *P* = 0.5849; amplitude estimate, 0.2592 *versus* 0.4595, *P* = 0.0004; and REM day 0 (BL) *versus* day 14: mesor estimate, 34.2977 *versus* 33.9672, *P* < 0.0001; acrophase estimate, 22.5019 *versus* 22.0298, *P* = 0.4794; amplitude estimate, 0.2592 *versus* 0.3713, *P* = 0.0347. *Blue rectangles* indicate circadian parameters that show statistically significant differences between day 0 and day 7 or day 14 in the SNI vehicle group. *Red rectangles* highlight the restoration or persistence of these differences in the SNI PGB group. *Purple rectangles* indicate the restoration or persistence of the differences observed in the SNI vehicle group, as well as additional significant differences uniquely induced in the SNI MOR group on both days 7 and 14. For SNI vehicle, *n* = 15; for SNI PGB, *n* = 7; and for SNI MOR, *n* = 8. *, *P* < 0.05; **, *P* < 0.01; ***, *P* < 0.001. BL, baseline; MOR, morphine; NREM, non–rapid eye movement; PGB, pregabalin; REM, rapid eye movement; SNI, spared nerve injury

The circadian rhythmicity of body temperature data was separately analyzed in different vigilance states during wakefulness, NREM sleep, and REM sleep. During wakefulness, SNI led to significant disruptions in both mesor and acrophase on day 7 and mesor disruption on day 14 (fig. 10A). Pregabalin treatment significantly reversed the acrophase disturbance on day 7, but the mesor remained impaired on both days (fig. 10B). Morphine also restored the acrophase on day 7 but introduced additional disruptions in the amplitude on both days (fig. 10C). During NREM sleep, both mesor and acrophase were significantly altered by SNI on days 7 and 14 (fig. 10D). Pregabalin significantly restored the acrophase on both days, although the mesor remained disrupted (fig. 10E). Morphine significantly restored both mesor and acrophase on day 7 but failed to maintain this improvement by day 14. Moreover, it induced amplitude disruptions on both days (fig. 10F). During REM sleep, SNI similarly disrupted mesor and acrophase parameters on both days 7 and 14 after SNI (fig. 10G). Pregabalin significantly restored the acrophase on both days but did not reverse the mesor abnormality (fig. 10H). Morphine restored the acrophase on days 7 and 14; however, it also introduced amplitude disruptions on both days (fig. 10I). Collectively, these findings indicate that pregabalin more consistently restores circadian rhythmicity of both locomotor activity and body temperature, particularly the acrophase parameter, across vigilance states, whereas morphine exerts partial benefits but introduces additional disturbances in circadian parameters, especially in amplitude.

### Diurnal Differences in Opioid-related Gene Expression in the Spinal Cord of Morphine-treated Mice

To investigate the effects of pregabalin on α_2_δ_1_ subunit gene expression and of morphine treatment on opioid-related gene expression in SNI mice, we analyzed the iSC samples for the expression of *Cacna2d1*, which encodes the α_2_δ_1_ auxiliary subunit of the voltage-gated Ca^2+^ channel (the primary target of pregabalin [α_2_δ_1_]), the endogenous opioid peptide precursors preproenkephalin (*Penk*) and prodynorphin (*Pdyn*), and opioid receptors δ (*Oprd1*), κ (*Oprk1*), and μ1 (*Oprm1*). To explore potential diurnal variations, samples were collected during the morning (9:00 to 11:00 am) and afternoon (2:00 to 4:00 pm) from both vehicle-treated and pregabalin- or morphine-treated SNI mice.

**Fig. 11.**
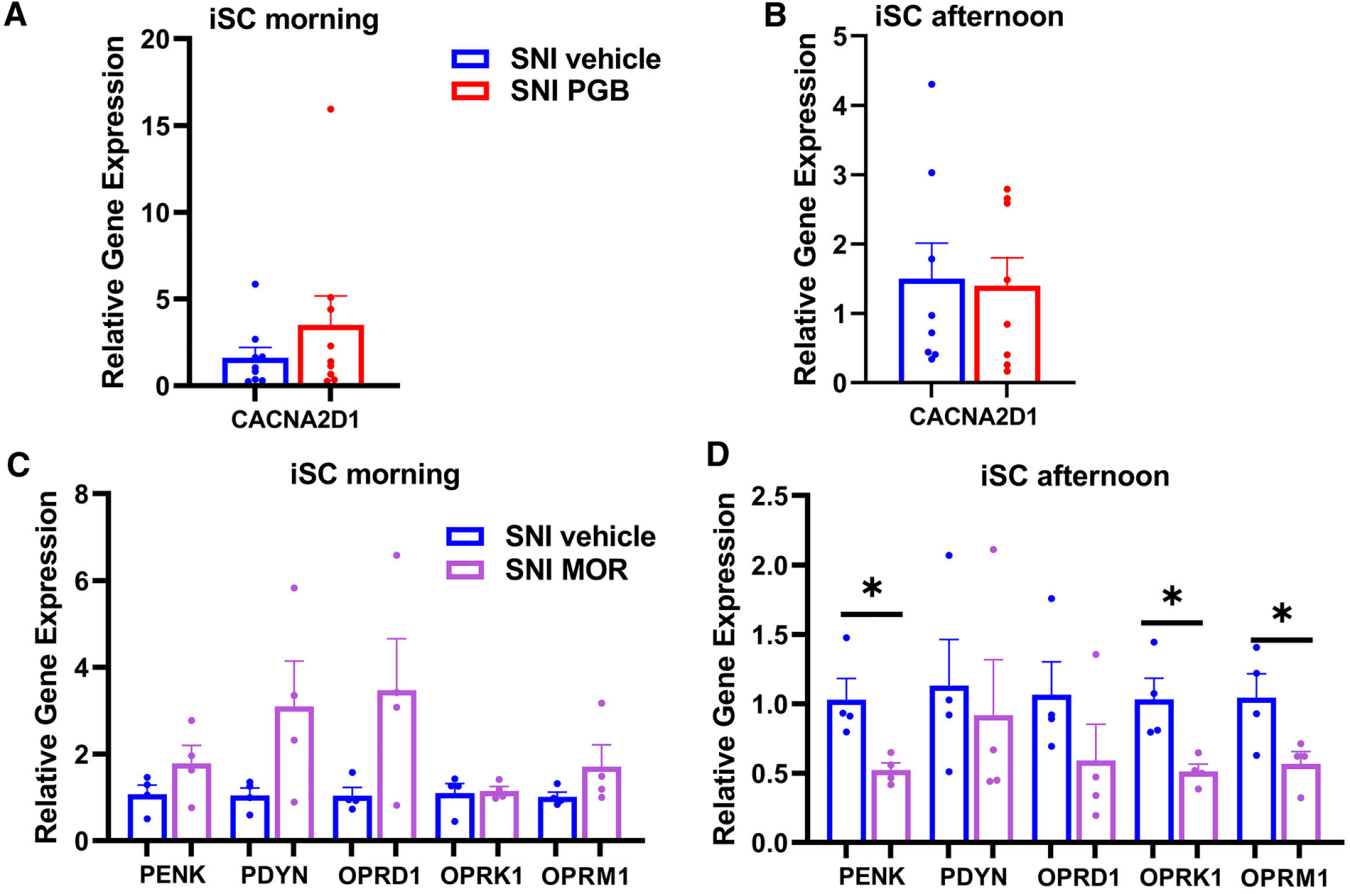
Diurnal changes in ipsilateral spinal cord expression of opioid-related genes in morphine-treated mice after spared nerve injury. (*A*) *Cacna2d1* expression in the iSC during the morning period (9:00 to 11:00 am) showed no significant differences between vehicle- and pregabalin-treated SNI mice. (*B*) *Cacna2d1* expression in the iSC during the afternoon period (2:00 to 4:00 pm) showed no significant differences between vehicle- and pregabalin-treated SNI mice. (*C*) *Penk*, *Pdyn*, *Oprd1*, *Oprk1*, and *Oprm1* expression in the iSC during the morning period (9:00 to 11:00 am) showed no significant differences between vehicle- and morphine-treated SNI mice. (*D*) In the afternoon (2:00 to 4:00 pm), *Penk*, *Oprk1*, and *Oprm1* expression levels were significantly reduced in morphine-treated SNI mice compared to vehicle-treated controls. For SNI vehicle *versus* SNI MOR in the afternoon: *Penk*, 1.029 ± 0.15 *versus* 0.5236 ± 0.05, *P* = 0.0197, 95% CI, −0.8983 to −0.1133; *Oprk1*, 1.031 ± 0.15 *versus* 0.5137 ± 0.05, *P* = 0.0184, 95% CI, −0.9119 to −0.1231; and *Oprm1*, 1.046 ± 0.17 *versus* 0.5694 ± 0.08, *P* = 0.0466, 95% CI, −0.9427 to −0.009779. Unpaired *t* tests were performed comparing SNI-PGB or SNI-MOR groups with the SNI-vehicle group at each time point. The data are presented as means ± SEM. *, *P* < 0.05. Sample sizes were as follows: for the morning session, for SNI vehicle, *n* = 9 (for *Cacna2d1*); for SNI PGB, *n* = 9; for SNI vehicle, *n* = 4 (for opioid-related genes); and for SNI MOR, *n* = 4; and for the afternoon session, for SNI vehicle, *n* = 8 (for *Cacna2d1*); for SNI PGB, *n* = 8; for SNI vehicle *n* = 4 (for opioid-related genes); and for SNI MOR, *n* = 4. iSC, ipsilateral spinal cord; MOR, morphine; PGB, pregabalin; SNI, spared nerve injury.

Our analysis revealed no significant differences in the expression of iSC *Cacna2d1* between vehicle- and pregabalin-treated mice in either morning or afternoon periods (fig. 11, A and B). Similarly, no significant differences were observed in the expression of iSC *Penk*, *Pdyn*, *Oprd1*, *Oprk1*, and *Oprm1* between vehicle- and morphine-treated SNI mice in the morning (fig. 11C). However, in the afternoon, the expression levels of *Penk*, *Oprk1*, and *Oprm1* were significantly reduced in morphine-treated SNI mice compared to vehicle-treated controls (fig. 11D).

### Differential Impacts of Analgesic Administration on Spinal Expression of Core Circadian Genes

To determine whether SNI disrupts the expression of core circadian genes in the iSC and whether pregabalin or morphine modulate these changes, we analyzed iSC samples collected during the morning (9:00 to 11:00 am) and afternoon (2:00 to 4:00 pm) from sham-operated mice, SNI mice, pregabalin-treated SNI mice, and morphine-treated SNI mice. Expression levels of seven core circadian genes, *Clock*, *Bmal1*, *Cry1*, *Cry2*, *Per1*, *Per2*, and *Per3*, were quantified and compared across the groups.

**Fig. 12.**
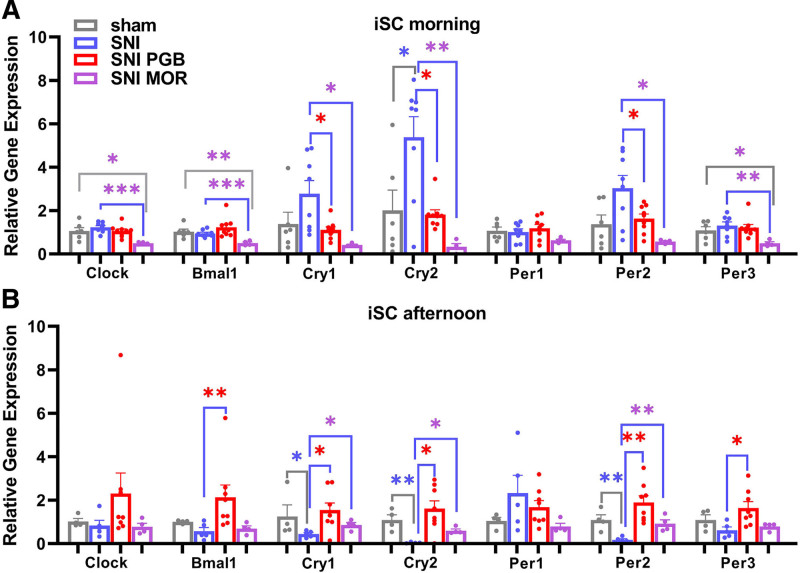
Diurnal changes in the expression of core circadian genes in the ipsilateral spinal cord after spared nerve injury and upon treatments. (*A*) In the morning period (9:00 to 11:00 am), SNI significantly increased the expression of *Cry2* compared to sham controls, which was reversed by pregabalin or morphine treatment. The increase in *Cry1* and *Per2* expression upon SNI was significantly reduced in pregabalin- or morphine-treated mice compared to SNI mice, with expression levels not significantly different from sham mice. In contrast, the expression of *Clock*, *Bmal1*, and *Per3* was further decreased in morphine-treated SNI mice compared to both SNI and sham controls. For sham *versus* SNI: *Cry2*, 2.015 ± 0.92 *versus* 5.381 ± 0.94, *P* = 0.0294, 95% CI, 0.4000 to 6.333; SNI *versus* SNI PGB: *Cry1*, 2.778 ± 0.60 *versus* 1.110 ± 0.14, *P* = 0.0122, 95% CI, −2.915 to −0.4206; and *Cry2*, 5.381 ± 0.94 *versus* 1.816 ± 0.22, *P* = 0.0111; *Per2*, 3.039 ± 0.57 *versus* 1.633 ± 0.20, *P* = 0.0302, 95% CI, −2.658 to −0.1544. For sham *versus* SNI PGB: *Cry1*, 1.383 ± 0.53 *versus* 1.110 ± 0.14, *P* = 0.9546; *Cry2*, 2.015 ± 0.92 *versus* 1.816 ± 0.22, *P* = 0.4559; and *Per2*, 1.372 ± 0.42 *versus* 1.633 ± 0.20, *P* = 0.5527, 95% CI, −0.6630 to 1.184. For SNI *versus* SNI MOR: *Cry1*, 2.778 ± 0.60 *versus* 0.4217 ± 0.03, *P* = 0.0224, 95% CI, −4.303 to −0.4093; *Cry2*, 5.381 ± 0.94 *versus* 0.3386 ± 0.14, *P* = 0.0044, 95% CI, −8.111 to −1.974; *Per2*, 3.039 ± 0.57 *versus* 0.5695 ± 0.04, *P* = 0.0148, 95% CI, −4.340 to −0.5988; *Per3*, 1.1312 ± 0.16 *versus* 0.4917 ± 0.07, *P* = 0.0068, 95% CI, −1.358 to −0.2828; *Clock*, 1.228 ± 0.08 *versus* 0.4985 ± 0.02, *P* = 0.0001, 95% CI, −0.9981 to −0.4619; and *Bmal1*, 0.9460 ± 0.05 *versus* 0.5022 ± 0.05, *P* = 0.0005, 95% CI, −0.6384 to −0.2490. For sham *versus* SNI MOR: *Cry1*, 1.383 ± 0.53 *versus* 0.4217 ± 0.33, *P* = 0.1143; *Cry2*, 2.015 ± 0.92 *versus* 0.3386 ± 0.14, *P* = 0.1889, 95% CI, −4.368 to 1.015; *Per2*, 1.372 ± 0.42 *versus* 0.5695 ± 0.04, *P* = 0.1698, 95% CI, −2.029 to 0.4241; *Per3*, 1.086 ± 0.17 *versus* 0.4917 ± 0.07, *P* = 0.0303, 95% CI, −1.116 to −0.07272; *Clock*, 1.060 ± 0.15 *versus* 0.4985 ± 0.02, *P* = 0.0223, 95% CI, −1.020 to −0.1032; and *Bmal1*, 1.030 ± 0.11 *versus* 0.5022 ± 0.05, *P* = 0.0086, 95% CI, −0.8806 to −0.1759. (*B*) In the afternoon (2:00 to 4:00 pm), *Cry1*, *Cry2*, and *Per2* expression levels were significantly reduced in SNI mice compared to sham controls. These reductions were reversed by pregabalin or morphine treatment, with expression levels not significantly different from sham levels. Additionally, pregabalin treatment significantly increased *Bmal1* and *Per3* expression compared to SNI mice and showed no significant difference compared to sham controls. For sham *versus* SNI: *Cry1*, 1.249 ± 0.53 *versus* 0.4443 ± 0.06, *P* = 0.0317; *Cry2*, 1.087 ± 0.23 *versus* 0.03996 ± 0.01, *P* = 0.0047, 95% CI, −1.632 to −0.4608; and *Per2*, 1.084 ± 0.23 *versus* 0.1757 ± 0.04, *P* = 0.0040, 95% CI, −1.420 to −0.3968. For SNI *versus* SNI PGB: *Cry1*, 0.4443 ± 0.06 *versus* 1.544 ± 0.32, *P* = 0.0241, 95% CI, 0.1734 to 2.027; *Cry2*, 0.03996 ± 0.01 *versus* 1.610 ± 0.35, *P* = 0.0130, 95% CI, 0.4094 to 2.731; *Per2*, 0.1757 ± 0.04 *versus* 1.890 ± 0.31, *P* = 0.0015, 95% CI, 0.8156 to 2.614; *Per3*, 0.6154 ± 0.15 *versus* 1.642 ± 0.28, *P* = 0.0233, 95% CI, 0.1682 to 1.884; and *Bmal1*, 0.5716 ± 0.16 *versus* 2.126 ± 0.56, *P* = 0.0062. For sham *versus* SNI PGB: *Cry1*, 1.249 ± 0.5361 *versus* 1.544 ± 0.32, *P* = 0.4606; *Cry2*, 1.087 ± 0.23 *versus* 1.610 ± 0.35, *P* = 0.3594, 95% CI, −0.6907 to 1.738; *Per2*, 1.084 ± 0.23 *versus* 1.890 ± 0.31, *P* = 0.1276, 95% CI, −0.2750 to 1.888; *Per3*, 1.086 ± 0.23 *versus* 1.642 ± 0.28, *P* = 0.2440, 95% CI, −0.4441 to 1.555; and *Bmal1*, 1.002 ± 0.03 *versus* 2.126 ± 0.56, *P* = 0.1535. For SNI *versus* SNI MOR: *Cry1*, 0.4443 ± 0.06 *versus* 0.8582 ± 0.11, *P* = 0.0127, 95% CI, 0.1193 to 0.7084; *Cry2*, 0.03469 ± 0.01 *versus* 0.5300 ± 0.07, *P* = 0.0286; and *Per2*, 0.1757 ± 0.04 *versus* 0.9175 ± 0.18, *P* = 0.0031, 95% CI, 0.3445 to 1.139. For sham *versus* SNI MOR: *Cry1*, 1.249 ± 0.5361 *versus* 0.8582 ± 0.11, *P* > 0.9999; *Cry2*, 1.087 ± 0.23 *versus* 0.5300 ± 0.07, *P* = 0.0571; and *Per2*, 1.084 ± 0.23 *versus* 0.9175 ± 0.18, *P* = 0.5972, 95% CI, −0.8977 to 0.5644. Unpaired *t* tests were performed comparing each treatment group (SNI PGB or SNI MOR) to SNI or sham controls, and comparing SNI to sham at each time point. *Gray lines* with *blue*, *red*, or *purple asterisks* denote significant differences between sham and SNI, sham and pregabalin-treated SNI, and sham and morphine-treated SNI mice, respectively. *Blue lines* with *red* or *purple asterisks* indicate significant differences between vehicle-treated SNI mice and pregabalin- or morphine-treated SNI mice, respectively. The data are presented as means ± SEM. *, *P* < 0.05; **, *P* < 0.01. Sample sizes were as follows: for the morning session: for sham, *n* = 6; for SNI, *n* = 8; for SNI PGB, *n* = 9; and for SNI MOR, *n* = 4; and for the afternoon session: for sham *n* = 4; for SNI, *n* = 5; for SNI PGB, *n* = 8; and for SNI MOR, *n* = 4. iSC, ipsilateral spinal cord; MOR, morphine; PGB, pregabalin; SNI, spared nerve injury.

In the morning, SNI mice showed a significant increase in *Cry2* expression compared to sham controls (fig. 12A). *Cry2* upregulation was reversed by both pregabalin and morphine, with expression returning to the level in sham mice for both treatment groups (fig. 12A). Similarly, *Cry1* and *Per2* expression were reduced by both pregabalin and morphine, with no significant differences observed between either treatment group and sham controls (fig. 12A). In contrast, the expression of *Clock*, *Bmal1*, and *Per3* was significantly decreased in morphine-treated SNI mice compared to both SNI mice and sham controls, indicating that morphine administration further disrupted the expression of these circadian genes (fig. 12A).

In the afternoon, significant downregulation of *Cry1*, *Cry2*, and *Per2* was observed in SNI mice compared to sham controls (fig. 12B). Treatment with either pregabalin or morphine restored the expression of these genes to levels not significantly different from sham mice (fig. 12B). Additionally, pregabalin significantly increased the expression of *Bmal1* and *Per3* compared to SNI mice, with expression of both genes restored to sham levels (fig. 12B).

Together, these results demonstrate that SNI disrupts the diurnal expression of core circadian genes in the spinal cord. This disruption was restored by pregabalin, whereas morphine showed mixed effects by ameliorating some of the changes and further disrupting others.

## Discussion

In the current study, we investigated the effects of pregabalin and morphine on neuropathic pain and associated disruptions in sleep architecture and circadian rhythms in a SNI mouse model. As main findings, pregabalin and morphine exert divergent effects on sleep and circadian regulation with neuropathic pain. Pregabalin improved sleep architecture by increasing REM sleep and reducing wakefulness, and enhanced sleep microstructure by increasing power density and spindle activity during sleep stage transitions. In contrast, morphine did not improve sleep and further dampened circadian rhythmicity. This is the first study to employ telemetric EEG for simultaneously monitoring sleep–wake architecture and circadian rhythms in freely moving mice with neuropathic pain, while evaluating the effects of continuous analgesics administration.

After SNI, REM sleep was reduced in both sexes compared to sham, confirmed by within-group comparisons to baseline. Given the essential role of γ-aminobutyric acid–mediated (GABAergic) neurons in REM sleep initiation and maintenance,^53,54^ this reduction may reflect neuropathic pain-induced disruptions in GABAergic signaling.^55^ Painful nerve injury in rodents leads to the loss of GABAergic neurons in the dorsal root ganglion and spinal cord,^55^ weakening pain inhibition and increasing nociceptive transmission to supraspinal structures involved in sleep regulation.^56^ Furthermore, presynaptic GABA release in the periaqueductal gray (PAG) was altered in rats with neuropathic pain.^57^ These disruptions in GABAergic signaling may alter sleep–wake regulation, potentially explaining the observed REM sleep reduction. As SNI reduced REM sleep in our study, we next explored the effects of pregabalin on restoring the sleep–wake cycle, since gabapentinoids have been shown to improve sleep quality in neuropathic pain patients by reducing sleep interference scores.^21,58,59^ Our results showed that pregabalin restored REM sleep to presurgical levels and reduced wakefulness. In contrast, morphine did not significantly affect the disturbed sleep architecture associated with neuropathic pain. Although no clinical studies have examined morphine’s effect on sleep in patients with neuropathic pain, its acute administration in healthy young adults reduces REM sleep, while increasing NREM stage 2 sleep.^27^ This suggests that the effects of morphine on sleep architecture may differ from those of pregabalin, potentially explaining its lack of efficacy in restoring REM sleep in our model. Within-group comparisons using non-normalized data showed that REM sleep was reduced in morphine-treated mice, indicating that morphine does not rescue REM sleep loss. Furthermore, we did not observe significant differences in NREM sleep between different treatment groups. However, within-group comparisons showed that NREM sleep progressively increased over time across all groups compared to baseline, suggesting that observed NREM sleep changes may reflect time-dependent physiologic adaptation rather than treatment effects.

Sleep spindles, the bursts of oscillatory brain activity visible on EEG during NREM sleep, play a critical role in sensory processing, memory consolidation, and synaptic plasticity.^52,60^ In our study, SNI-vehicle mice exhibited increased spindle activity during transitions between wakefulness and NREM, which may indicate increased sleep instability or fragmentation. Pregabalin increased spindle counts during transitions between NREM and REM sleep, while reducing the transitions between NREM and wakefulness, suggesting enhanced sleep stability and architecture.^52,61^ In contrast, morphine did not affect transitions between sleep stages. Increased spindle activity during these transitions is linked to enhanced memory consolidation and cognitive function.^52,62,63^ Neuropathic pain impairs cognition. For example, patients with chronic neuropathic or radicular pain exhibit impairments in spatial and verbal memory and altered attentional responses.^64,65^ Preclinical studies support these findings, showing that neuropathic pain due to chronic constriction injury leads to cognitive impairment in mice.^66,67^ Additionally, the glymphatic system, a brain waste clearance mechanism highly active during NREM sleep, may play a complementary role in pregabalin’s effects on sleep and cognition. Enhanced sleep spindle activity could promote more efficient glymphatic function, facilitating neurotoxic waste clearance.^68–70^ This may be particularly relevant in neuropathic pain, where neuroinflammation and oxidative stress exacerbate cognitive decline.^71^ Interestingly, benzodiazepines like zolpidem, commonly used for insomnia, reduce neurovascular and neuronal oscillatory dynamics, thereby diminishing glymphatic flow.^68^ In contrast, pregabalin may enhance glymphatic activity indirectly through its modulation of sleep architecture, which warrants further investigation. In addition to direct sleep effects, pregabalin might also improve sleep quality by reducing anxiety.^72,73^ A meta-analysis of clinical studies reported that 53% of the effects of pregabalin on sleep disturbance was direct, while 47% was mediated by anxiety reduction.^72^

Our results also revealed that pregabalin increased EEG power spectra (3.5 to 5.5 Hz) during REM sleep in SNI mice, particularly within the delta (1 to 4 Hz) and low theta (4 to 6 Hz) bands, compared to vehicle. This is noteworthy, as theta oscillations are a hallmark of REM sleep in mice and are critical for memory consolidation and emotional regulation.^74^ The persistence of these effects up to 14 days after injury suggests a prolonged, potentially cumulative effect of pregabalin on REM sleep. In contrast, morphine-treated mice showed no significant alterations in REM sleep power spectra, highlighting the distinct mechanisms of pregabalin, possibly involving modulation of GABAergic and glutamatergic neurotransmission,^75,76^ both essential for REM sleep regulation.^77–80^

Using cosinor analysis, we assessed the effects of pregabalin and morphine on circadian parameters of mesor, amplitude, and acrophase.^81^ Results showed that mesor, a measure of the rhythm’s average level, was disrupted in SNI mice on both days 7 and 14 after injury. This disruption is consistent with previous studies showing neuropathic pain disrupts circadian rhythms.^14,82,83^ Pregabalin treatment significantly improved circadian rhythmicity of locomotor activity, restoring the mesor on both days 7 and 14 after injury, suggesting that pregabalin has a stabilizing effect on circadian rhythms, potentially mediated through its modulation of GABAergic neurotransmission.^84^ GABAergic neurons play a crucial role in maintaining circadian rhythms by regulating the activity of the suprachiasmatic nucleus, the master circadian clock in mammals.^85^ In contrast, while morphine also improved the mesor, it disrupted the acrophase on day 7 after injury. The acrophase, the timing of peak activity within the circadian cycle, is crucial for synchronizing physiologic processes.^14,86^ Previous studies have shown that opioids can disrupt the integrity and timing of the circadian system,^87^ both in therapeutic contexts and in cases of disordered opioid use. Moreover, disruptions in the circadian system can alter the efficacy and adverse effects of opioids.^88^ The circadian rhythm of body temperature was also evaluated, revealing disruptions in both the mesor and acrophase after SNI. Pregabalin treatment effectively restored the acrophase on both days 7 and 14, indicating a normalization of the timing of peak body temperature. However, the mesor remained disrupted, suggesting that while pregabalin can correct the timing of circadian peaks, it may not fully restore the overall average level of body temperature. This partial restoration aligns with the role of pregabalin in modulating calcium channels and neurotransmitter release in the central nervous system, which are crucial for maintaining circadian homeostasis.^18,89^ In contrast, morphine also restored the acrophase but introduced a new disruption in the amplitude of the body temperature rhythm, indicating an inconsistent effect on circadian regulation. Amplitude, representing the range of variation within a circadian cycle, is critical for robust and synchronized physiologic rhythms.^90^

To evaluate the diurnal changes in the spinal expression of downstream targets of morphine^91–94^ and pregabalin^17–19^ upon SNI, we measured the expression of δ- (*Oprd1*), κ- (*Oprk1*), μ- (*Oprm1*) opioid receptor, preproenkephalin (*Penk*), and prodynorphin (*Pdyn*) in morphine-treated mice and expression of α_2_δ_1_ (*Cacna2d1*) in pregabalin-treated mice. Analysis revealed significant diurnal variations in opioid-related gene expression, while spinal expression of α_2_δ_1_ in pregabalin-treated mice showed no diurnal change *versus* controls. In the afternoon, expression levels of preproenkephalin (*Penk*), κ- (*Oprk1*), and μ-opioid receptor (*Oprm1*) were significantly decreased in morphine-treated SNI mice, compared to controls. A previous study in sciatic nerve ligation mice reported rhythmic changes in the expression of μ- and κ-opioid receptors in the PAG.^95^ To further explore the impact of SNI and analgesic treatment on circadian regulation, we examined the spinal expression of core circadian genes. SNI disrupted the diurnal expression of *Cry1*, *Cry2*, and *Per2*, consistent with our recent findings of hundreds of circadian transcriptome alterations in the iSC and PAG upon SNI.^83^ Additionally, while pregabalin and morphine were able to restore the circadian gene expression upon SNI, morphine further disrupted the expression of *Clock* and *Per3*, in line with evidence indicating adverse circadian effects of morphine treatment.^27–29^

Our study has limitations. While neuropathic pain is more prevalent in the elderly,^96^ we used young adult mice to minimize age-related variability in outcomes. Given that aging is associated with reduced and fragmented sleep, as well as dampened circadian rhythms,^97^ future studies should include aged mice to enhance translational relevance. Additionally, due to sex differences in pain perception and analgesic efficacy, women often exhibit higher drug concentrations and increased susceptibility to side effects from neuropathic pain medications^98^; future studies should include female mice.

In conclusion, our results suggest that pregabalin may offer distinct advantages over morphine in treating neuropathic pain, particularly in restoring disrupted sleep architecture and stabilizing circadian rhythms. Although both analgesics relieved pain, only pregabalin significantly enhanced REM sleep, sleep spindles, and circadian rhythms. These findings highlight the importance of considering circadian dynamics when developing treatment strategies for neuropathic pain. Optimizing dosing times according to circadian principles could enhance efficacy and reduce side effects, offering new directions for clinical pain management.

### Acknowledgments

The authors thank Dr. Pekka Rauhala (Department of Pharmacology, University of Helsinki, Helsinki, Finland) for kindly providing the drugs used in this study, Dr. James M. McNally (Department of Psychiatry, Harvard Medical School, Boston, Massachusetts) for kindly providing the MATLAB-based script for analysis of sleep spindles, and Les Hearn, M.Sc., for proofreading the manuscript.

### Research Support

Supported by grant No. 341475 from the Research Council of Finland (Helsinki, Finland; to Dr. Palada) and by funds from the Päivikki and Sakari Sohlberg Foundation (Helsinki, Finland; to Dr. Palada), the Liv och Hälsa Foundation (Helsinki, Finland; to Dr. Palada), the John J. Bonica Fellowship from the International Association for the Study of Pain (Washington, D.C.; to Dr. Palada), and the Finnish Medical Association, Finska Läkaresällskapet (Helsinki, Finland; to Dr. Kalso).

### Competing Interests

The authors declare no competing interests.

## Supplemental Digital Content

Supplemental Fig. S1. Recovery from EEG implantation in protocol 1, https://links.lww.com/ALN/E179

Supplemental Fig. S2. Reduction of non-normalized REM epochs upon SNI, https://links.lww.com/ALN/E180

Supplemental Fig. S3. Recovery from EEG implantation in protocol 2, https://links.lww.com/ALN/E181

Supplemental Fig. S4. Pregabalin restores non-normalized REM epochs, https://links.lww.com/ALN/E182

Supplemental Fig. S5. Effect of drugs on spindle counts in transitions, https://links.lww.com/ALN/E183

## Supplementary Material
