# Supplementary material for: Differential Effects of Pregabalin and Morphine on the Sleep–Wake Cycle and Circadian Rhythms in Mice with Neuropathic Pain
Source: Anesthesiology. 2025 Aug 13;143(5):1313–39. doi: 10.1097/ALN.0000000000005715 (PMC12513049; doi:10.1097/ALN.0000000000005715)
Supplement: Supplementary file 1 [file aln-143-1313-s001.pdf]

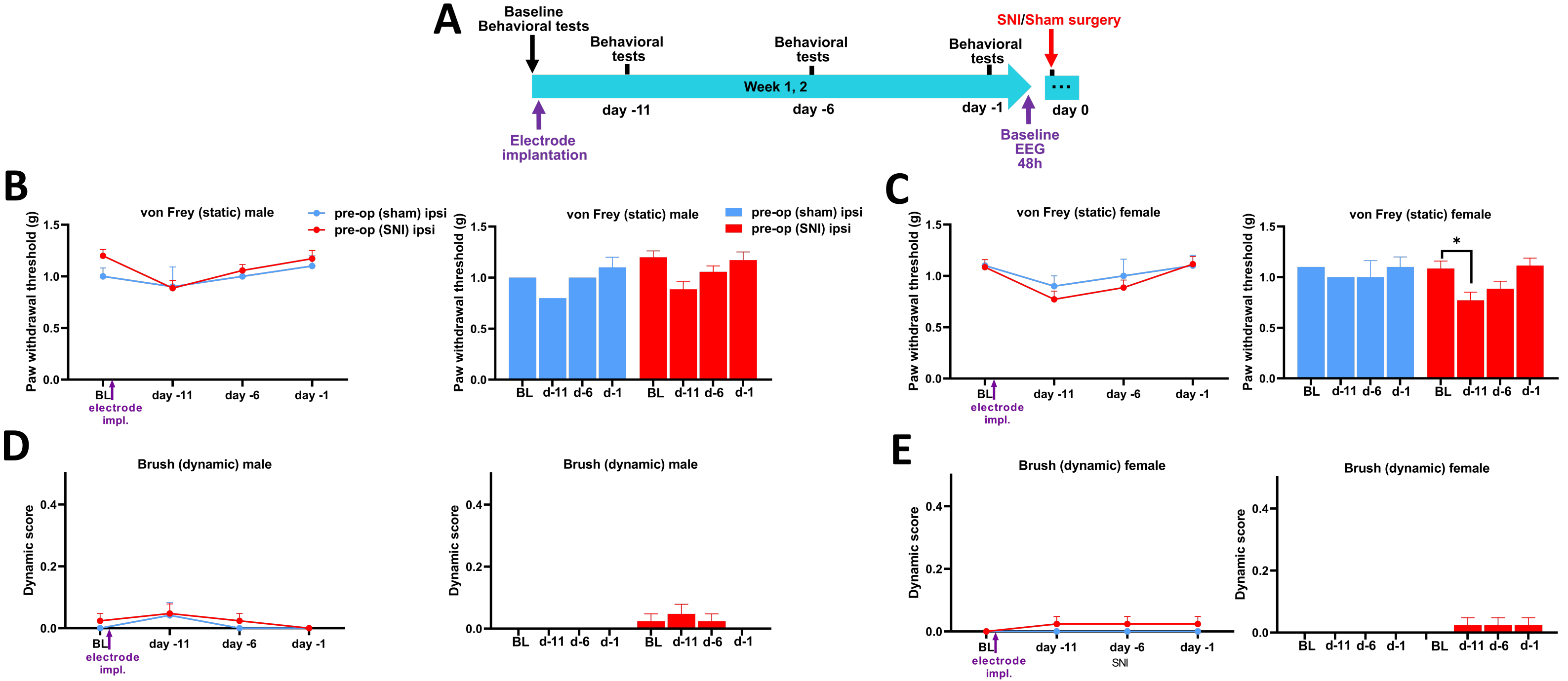

A
Baseline
Behavioral tests
Behavioral tests
Behavioral tests
Behavioral tests
SNI/Sham surgery
Week 1, 2
day -11
day -6
day -1
day 0
Electrode implantation
Baseline EEG 48h
B
von Frey (static) male
pre-op (sham) ipsi
pre-op (SNI) ipsi
Paw withdrawal threshold (g)
BL
day -11
day -6
day -1
electrode impl.
d-11
d-6
d-1
C
von Frey (static) female
*
D
Brush (dynamic) male
Dynamic score
E
Brush (dynamic) female
SNI

Fig. S1: Assessment of mechanical allodynia during the 2-week recovery period following EEG electrode implantation in mice from protocol 1. (A) Schematic diagram of the experimental procedure during the recovery period, adapted from the full protocol in Fig. 1B, showing the timeline from baseline behavioral testing, EEG transmitter implantation, and recovery period, up to SNI or sham surgery (defined as day 0). Behavioral tests were performed on days 3, 8, and 13 post-electrode implantation, corresponding to day -11, day -6, and day -1 in the full protocol timeline. **(B)** Paw withdrawal thresholds to von Frey filament stimulations in male mice undergoing EEG transmitter implantation. Right panel: comparison of threshold changes at each post-implantation time point to the respective baseline (BL) values. A decrease trend (P =0.0625) in withdrawal threshold was observed for post-implantation day 3 (day -11), and no significant differences were found on days 8, and 13 post-implantation (days -6, and -1 respectively). **(C)** Paw withdrawal thresholds to von Frey filament stimulations in female mice undergoing EEG transmitter implantation. Right panel: comparison of thresholds changes at each post-implantation time point to the respective baseline values. A significant reduction in withdrawal threshold was observed on day 3 post-implantation (day -11), while no significant changes were found on days 8 and 13 post-implantation (days -6 and -1 respectively). **(D)** Dynamic scores in response to brush dynamic test in male mice undergoing EEG transmitter implantation. Right panel: comparisons of each post-implantation time point to the baseline values. No significant differences were observed. **(E)** Dynamic scores in response to brush stimulations in female mice undergoing EEG transmitter implantation. Right panel: comparisons of each time point with baseline values. No significant differences were detected. Paired t-tests were performed for each group with their BL, for each time point. Data are presented as mean ± SEM, * $P < 0.05$. pre-op (sham) $n = 4$ per sex, pre-op (SNI) $n = 7$ per sex. (In the figure, SNI = spared nerve injury; BL = baseline; ipsi = ipsilateral, pre-op = pre-operation (i.e., before SNI/sham surgeries), impl. = implantation.)
