# Supplementary material for: Differential Effects of Pregabalin and Morphine on the Sleep–Wake Cycle and Circadian Rhythms in Mice with Neuropathic Pain
Source: Anesthesiology. 2025 Aug 13;143(5):1313–39. doi: 10.1097/ALN.0000000000005715 (PMC12513049; doi:10.1097/ALN.0000000000005715)
Supplement: Supplementary file 2 [file aln-143-1313-s002.pdf]

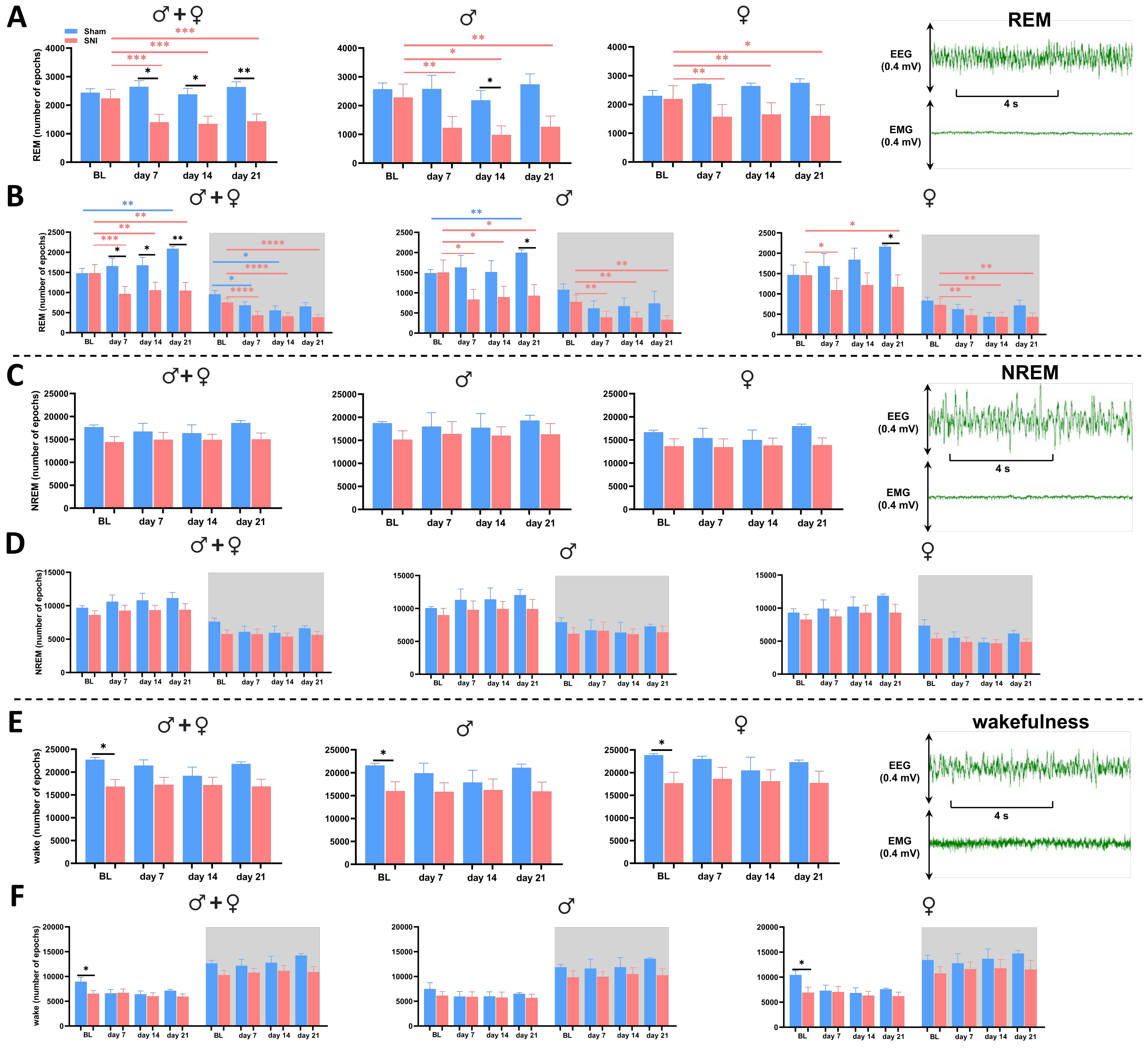
A
♂+♀
♂
♀
Sham
SNI
REM (number of epochs)
BL
day 7
day 14
day 21
REM
EEG (0.4 mV)
EMG (0.4 mV)
4 s
B
REM (number of epochs)
C
NREM (number of epochs)
NREM
D
E
wake (number of epochs)
wakefulness
F

**Fig. S2.: Spared nerve injury reduces the number of non-normalized REM sleep epochs. (A)** The number of REM sleep epochs was assessed on days 7, 14, and 21 after SNI. A significant reduction in REM sleep epochs was observed from day 7 and persisted through day 21 post-surgery, in the combined data for both sexes and in male mice on day 14 post-SNI. The same reduction in REM sleep epochs was also observed when compared to the respective baseline levels for the combined data and male and female mice on post-surgical days 7, 14 and 21 (left to right). The rightmost panel shows an example of EEG/EMG signals in REM sleep. **(B)** A reduction in REM sleep epochs during the light phase was observed in SNI mice from day 7 to day 21 in the combined data, and on day 21 in male and female SNI mice analysed separately, compared to sham controls (left to right). The same reduction in REM sleep epochs were also observed within-groups when compared to respective baseline levels, for the combined data and male and female mice analysed separately, during both the light and dark phases on days 7, 14, and 21, in the combined data as well as in males and females separately, when compared to baseline (left to right). **(C)** The number of NREM sleep epochs was assessed on days 7, 14, and 21 post-SNI. No significant differences in NREM sleep epochs were observed in the combined data for both sexes, as well as in male and female mice analysed separately (left to right). The rightmost panel shows an example of EEG/EMG signals in NREM sleep. **(D)** No significant differences in NREM sleep were observed during either the light or dark phases in the combined data, or in male and female mice analysed separately (left to right). **(E)** The number of wakefulness epochs was assessed on days 7, 14, and 21 post-SNI. Significant differences were observed between sham and SNI groups at baseline for both sexes. The rightmost panel shows an example of EEG/EMG signals in wakefulness. **(F)** A significant difference in the number of wakefulness epochs during the light phase at baseline was observed in the combined data and in female mice, when comparing sham and SNI groups. Black asterisks (*) and lines indicate significant differences between sham and SNI groups; Blue asterisks (*) and lines indicate significant within-group differences between days 7, 14, or 21 and baseline in the sham group; Pink asterisks (*) and lines indicate significant within-group differences between day 7, 14, or 21 and baseline in the SNI group. Unpaired *t*-tests were used for comparisons between SNI and sham groups at each time point. Paired t-tests were used for within-group comparisons between post-surgical days and baseline. Data are presented as mean ± SEM, EEG sham $n$ = 4/sex, EEG SNI $n$ =

7/sex. * $P < 0.05$, ** $P < 0.01$, *** $P < 0.001$, **** $P < 0.0001$. (In the figure, BL = baseline; SNI = spared nerve injury.)
