# Supplementary material for: Differential Effects of Pregabalin and Morphine on the Sleep–Wake Cycle and Circadian Rhythms in Mice with Neuropathic Pain
Source: Anesthesiology. 2025 Aug 13;143(5):1313–39. doi: 10.1097/ALN.0000000000005715 (PMC12513049; doi:10.1097/ALN.0000000000005715)
Supplement: Supplementary file 3 [file aln-143-1313-s003.pdf]

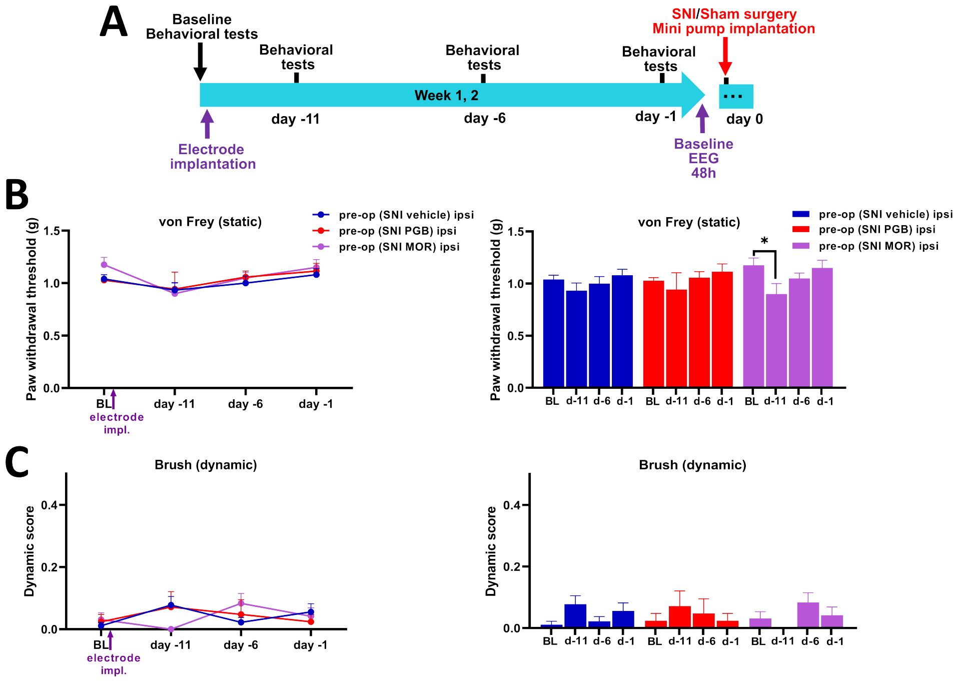

A
Baseline
Behavioral tests
Behavioral
tests
Behavioral
tests
Behavioral
tests
SNI/Sham surgery
Mini pump implantation
Week 1, 2
day -11
day -6
day -1
day 0
Electrode
implantation
Baseline
EEG
48h
B
von Frey (static)
Paw withdrawal threshold (g)
pre-op (SNI vehicle) ipsi
pre-op (SNI PGB) ipsi
pre-op (SNI MOR) ipsi
BL
day -11
day -6
day -1
electrode
impl.
BL d-11 d-6 d-1
C
Brush (dynamic)
Dynamic score

Fig. S3: Mechanical allodynia assessment during the 2-week recovery period following EEG electrode implantation in mice for protocol 2. (A) Schematic diagram of the experimental procedure during the recovery period, adapted from the full protocol in Fig. 1C, illustrating the timeline from baseline behavioral testing, EEG transmitter implantation, and recovery, up to SNI or sham surgery (defined as day 0). Behavioral tests were performed on days 3, 8, and 13 post-electrode implantation, corresponding to day -11, day -6, and day -1 in the full protocol timeline, respectively. **(B)** Paw withdrawal thresholds to von Frey filament stimulations in mice later assigned to vehicle, pregabalin, or morphine treatment groups. Right panel: Compared to respective baseline values, a significant decrease in withdrawal threshold was detected on day 3 post-implantation (day -11) in the pre-op (SNI MOR) group. No significant differences were found on days 8 and 13 post implantation (days -6, and -1, respectively). **(G)** Dynamic scores in response to brush dynamic test in mice which were later assigned to vehicle, pregabalin, or morphine treatment groups across the post-implantation time points (days -11, -6, and -1). Right panel: Comparison to baseline values indicated no significant differences. Paired t-tests were performed for each group with their baseline values at each time point. Data are presented as mean ± SEM, * $P < 0.05$. pre-op (SNI vehicle) $n = 15$, pre-op (SNI PGB) $n = 7$, pre-op (SNI MOR) $n = 8$. (In the figure, SNI = spared nerve injury; PGB = pregabalin; MOR = morphine; BL = baseline; ipsi = ipsilateral, pre-op = pre-operation (i.e., before SNI/sham surgeries), impl. = implantation.)
