# Supplementary material for: Differential Effects of Pregabalin and Morphine on the Sleep–Wake Cycle and Circadian Rhythms in Mice with Neuropathic Pain
Source: Anesthesiology. 2025 Aug 13;143(5):1313–39. doi: 10.1097/ALN.0000000000005715 (PMC12513049; doi:10.1097/ALN.0000000000005715)
Supplement: Supplementary file 4 [file aln-143-1313-s004.pdf]

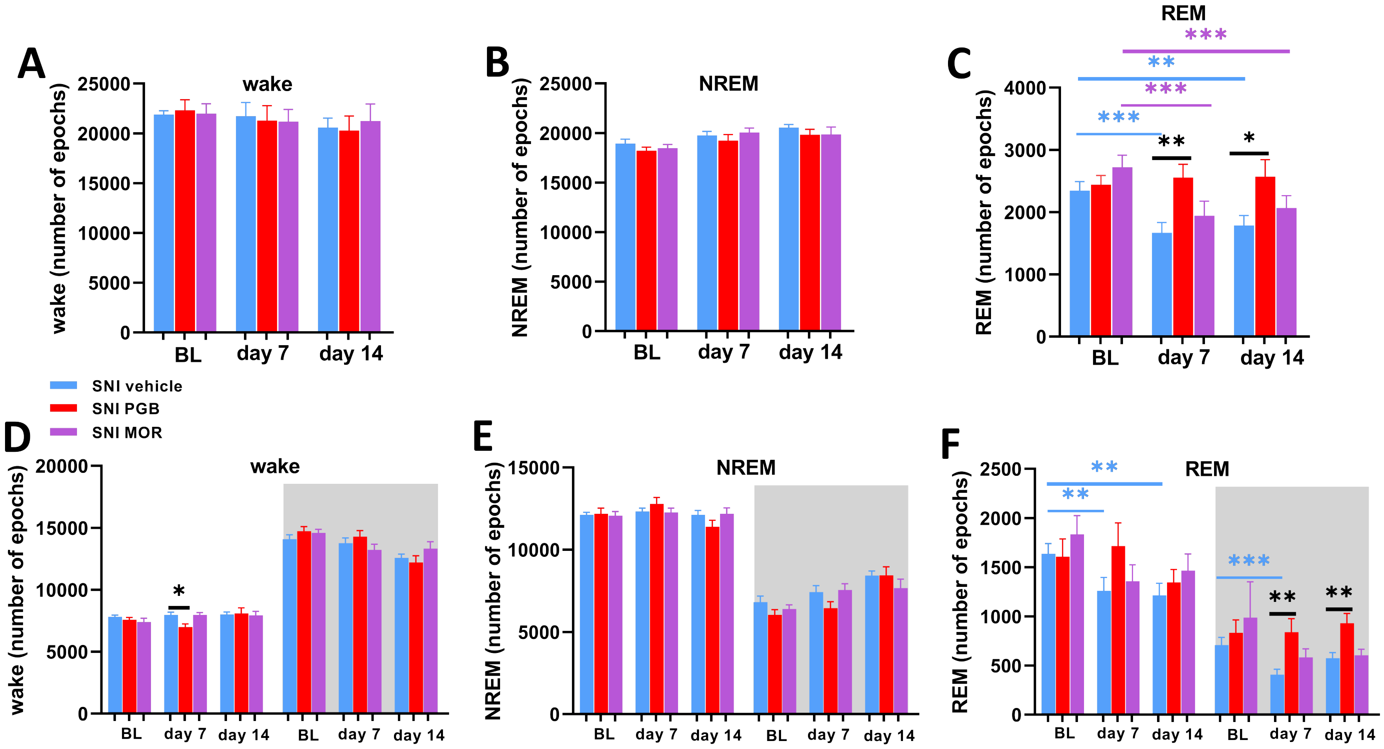

A
wake
wake (number of epochs)
25000
20000
15000
10000
5000
0
BL
day 7
day 14
B
NREM
NREM (number of epochs)
C
REM
REM (number of epochs)
4000
3000
2000
1000
***
**
*
SNI vehicle
SNI PGB
SNI MOR
D
E
F
15000
2500
1500
500

Fig. S4.: Continuous administration of pregabalin restores non-normalized REM sleep epochs number to pre-surgical levels after spared nerve injury. EEG recordings were performed at baseline before SNI and on day 7 and 14 post-SNI, for 48 hours at each time point. Data were analyzed as 4-second epochs for each sleep-wake stage. **(A)** No significant differences in wakefulness were observed between pregabalin- or morphine-treated SNI mice and vehicle-treated SNI mice. In addition, no significant changes in wakefulness were found within each treatment group compared to their own baseline levels at either day 7 or day 14. **(B)** Neither pregabalin nor morphine significantly affected NREM sleep in SNI mice. **(C)** REM sleep epochs were significantly increased on days 7 and 14 post-SNI in pregabalin-treated mice, compared to vehicle-treated SNI mice. In contrast, morphine-treated SNI mice showed no significant difference in REM sleep compared to the vehicle group. Within-group comparisons showed that the number of REM sleep epochs was significantly reduced in vehicle-treated SNI mice on both days 7 and 14 compared to baseline, and a similar reduction was observed in morphine-treated mice. In contrast, no significant difference in REM sleep was found in pregabalin-treated mice relative to their baseline levels. **(D)** Wakefulness epochs were also analyzed separately for light and dark phases. A significant reduction in wakefulness was observed during the light phase on day 7 post-SNI in pregabalin-treated mice, compared to vehicle-treated mice. **(E)** No significant differences in NREM sleep were observed during either the light or dark phase in pregabalin- or morphine-treated mice, compared to the vehicle group. **(F)** REM sleep epochs were significantly increased in pregabalin-treated mice during the dark phase on both days 7 and 14, compared to vehicle-treated mice. No significant differences in REM sleep were found in morphine-treated mice during either phase. Furthermore, within-group analysis showed a significant reduction in REM sleep epochs in vehicle-treated mice on both days 7 and 14 during the light phase, and on day 7 during the dark phase, compared to baseline. Unpaired t-tests were performed to compare SNI PGB and SNI vehicle groups, as well as SNI MOR and SNI vehicle groups, for each time point. Paired t-tests were used to compare each treatment group with its own baseline values. Data are presented as mean ± SEM. SNI vehicle n = 15, SNI PGB n = 7, SNI MOR n = 8, * $P < 0.05$, ** $P < 0.01$. (In the figure, BL = baseline; SNI = spared nerve injury; PGB = pregabalin; MOR = morphine)
