# Supplementary material for: Differential Effects of Pregabalin and Morphine on the Sleep–Wake Cycle and Circadian Rhythms in Mice with Neuropathic Pain
Source: Anesthesiology. 2025 Aug 13;143(5):1313–39. doi: 10.1097/ALN.0000000000005715 (PMC12513049; doi:10.1097/ALN.0000000000005715)
Supplement: Supplementary file 5 [file aln-143-1313-s005.pdf]

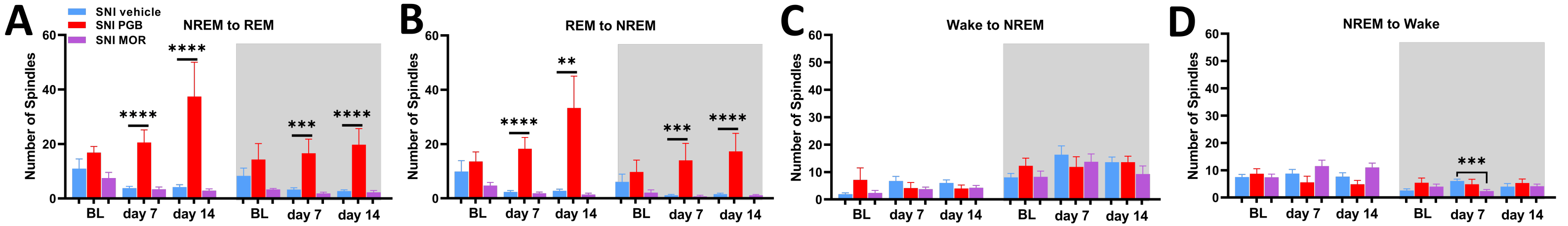
A
SNI vehicle
SNI PGB
SNI MOR
NREM to REM
Number of Spindles
0
20
40
60
****
****
***
****
BL
day 7
day 14
BL
day 7
day 14
B
REM to NREM
Number of Spindles
0
20
40
60
****
**
***
****
BL
day 7
day 14
BL
day 7
day 14
C
Wake to NREM
Number of Spindles
0
10
20
30
40
50
60
BL
day 7
day 14
BL
day 7
day 14
D
NREM to Wake
Number of Spindles
0
10
20
30
40
50
60
***
BL
day 7
day 14
BL
day 7
day 14

**Fig. S5.: Sleep spindle counts are increased during NREM and REM sleep transitions in pregabalin-treated mice. (A)** During the transition from NREM to REM sleep, pregabalin-treated mice exhibited significantly increased spindle counts in both the light and dark phases on days 7 and 14 post-SNI, compared to vehicle-treated mice. No significant changes were observed in morphine-treated mice. **(B)** Similarly, during the transition from REM to NREM sleep, spindle counts were significantly elevated in pregabalin-treated mice across both phases and both time points (days 7 and 14 post-SNI), whereas no significant differences were found in morphine-treated mice, compared to vehicle-treated controls. **(C)** No significant differences in spindle counts were observed during the transition from wakefulness to NREM sleep in either pregabalin- or morphine-treated mice, compared to vehicle-treated SNI mice. **(D)** During the transition from NREM sleep to wakefulness, spindle counts were significantly decreased in morphine-treated mice during the dark phase on day 7 post-SNI, compared to vehicle-treated mice. No significant changes were found in the pregabalin group. Unpaired *t*-tests were performed between the SNI PGB and SNI vehicle groups, and between the SNI MOR and SNI vehicle groups, at each time point. Data are presented as mean ± SEM. SNI vehicle $n$ = 15, SNI PGB $n$ = 7, SNI MOR $n$ = 8. ** $P < 0.01$, *** $P < 0.001$, **** $P < 0.0001$. (In the figure, BL = baseline; SNI = spared nerve injury; PGB = pregabalin; MOR = morphine).
